# A global sensitivity analysis of a mechanistic model of neoadjuvant chemotherapy for triple negative breast cancer constrained by in vitro and in vivo imaging data

**DOI:** 10.1007/s00366-023-01873-0

**Published:** 2023-08-07

**Authors:** Guillermo Lorenzo, Angela M. Jarrett, Christian T. Meyer, Julie C. DiCarlo, John Virostko, Vito Quaranta, Darren R. Tyson, Thomas E. Yankeelov

**Affiliations:** 1 Department of Civil Engineering and Architecture, University of Pavia, Via Ferrata 3, 27100 Pavia, Italy; 2 Oden Institute for Computational Engineering and Sciences, The University of Texas at Austin, Austin, USA; 3 Livestrong Cancer Institutes, Dell Medical School, The University of Texas at Austin, Austin, USA; 4 Center for Cancer Systems Biology at Vanderbilt, Vanderbilt University, Nashville, USA; 5 Duet BioSystems, Inc., Nashville, USA; 6 Biomedical Imaging Center, The University of Texas at Austin, Austin, USA; 7 Department of Diagnostic Medicine, The University of Texas at Austin, Austin, USA; 8 Department of Oncology, The University of Texas at Austin, Austin, USA; 9 Department of Biochemistry, Vanderbilt University, Nashville, USA; 10 Department of Pharmacology, Vanderbilt University School of Medicine, Nashville, USA; 11 Department of Biomedical Engineering, The University of Texas at Austin, Austin, USA; 12 Department of Imaging Physics, The University of Texas MD Anderson Cancer Center, Houston, USA

**Keywords:** Magnetic resonance imaging, Time-resolved microscopy, Computational oncology, Breast cancer, Sensitivity analysis, Isogeometric analysis

## Abstract

Neoadjuvant chemotherapy (NAC) is a standard-of-care treatment for locally advanced triple negative breast cancer (TNBC) before surgery. The early assessment of TNBC response to NAC would enable an oncologist to adapt the therapeutic plan of a non-responding patient, thereby improving treatment outcomes while preventing unnecessary toxicities. To this end, a promising approach consists of obtaining in silico personalized forecasts of tumor response to NAC via computer simulation of mechanistic models constrained with patient-specific magnetic resonance imaging (MRI) data acquired early during NAC. Here, we present a new mechanistic model of TNBC growth and response to NAC, including an explicit description of drug pharmacodynamics and pharmacokinetics. As longitudinal in vivo MRI data for model calibration is limited, we perform a sensitivity analysis to identify the model mechanisms driving the response to two NAC drug combinations: doxorubicin with cyclophosphamide, and paclitaxel with carboplatin. The model parameter space is constructed by combining patient-specific MRI-based in silico parameter estimates and in vitro measurements of pharmacodynamic parameters obtained using time-resolved microscopy assays of several TNBC lines. The sensitivity analysis is run in two MRI-based scenarios corresponding to a well-perfused and a poorly perfused tumor. Out of the 15 parameters considered herein, only the baseline tumor cell net proliferation rate along with the maximum concentrations and effects of doxorubicin, carboplatin, and paclitaxel exhibit a relevant impact on model forecasts (total effect index, $S_{T}$0.1). These results dramatically limit the number of parameters that require in vivo MRI-constrained calibration, thereby facilitating the clinical application of our model.

**Supplementary Information:**

The online version contains supplementary material available at 10.1007/s00366-023-01873-0.

## Introduction

Neoadjuvant chemotherapy (NAC) is widely considered a standard-of-care treatment for stage II–III, locally advanced triple negative breast cancer (TNBC) prior to surgery [1–3]. NAC usually consists of one or two consecutive chemotherapeutic regimens delivered over the course of 4–6 months. Each of these regimens may involve one or two cytotoxic drugs, which induce tumor cell death (e.g., doxorubicin, paclitaxel). Hence, the use of NAC in TNBC patients aims to reduce the tumor size, which increases the success rate of breast conservation surgery [4–6]. NAC may also completely eliminate the tumor, an outcome which is known as a pathological complete response (pCR). Importantly, patients who achieve pCR in the neoadjuvant setting are known to have a significantly better prognosis and recurrence-free survival, whereas patients who have residual disease after NAC are at increased risk of early recurrence and death [7–9]. Thus, the early determination of response to NAC would enable the treating oncologist to adapt the therapeutic regimen of a non-responding patient (e.g., by changing the prescribed drugs as well as their dosage and schedule). This treatment adjustment could improve therapeutic outcomes while avoiding unnecessary toxicities [2, 3, 10, 11]. In particular, the early optimization of NAC would be especially advantageous in improving the therapeutic success in TNBC, which usually shows an adverse prognosis and is difficult to treat effectively [3, 12]. Additionally, accurately predicting response to NAC would enable identification of exceptional responders who might benefit from treatment de-escalation, including the possibility of non-surgical management of their disease [13–16].

To definitively establish that switching therapy significantly improves patient outcomes, accurate methods for predicting response early in the course of NAC are required. The existing approaches usually consist of either imaging-based changes in tumor size metrics [17, 18] or tissue-based biomarkers [19–22]. However, tumor size-based methods cannot definitively establish changes until the patient has received several treatment cycles [23, 24] and, since tissue-based biomarkers require an invasive biopsy, they are prone to sampling errors due to tumor heterogeneity [25–27]. Alternatively, imaging-based computational tumor forecasting has been actively investigated to obtain early predictions of patient-specific pathological and therapeutic outcomes that can guide clinical decision-making for different tumor types [28–37]. These tumor forecasting methods align with other imaging-based predictive technologies that have been applied to multiple pathologies within the context of computational medicine [38–43]. In particular, spatiotemporally-resolved computational forecasts of breast cancer response to NAC have shown promise in predicting the therapeutically-induced reduction of tumor burden at the conclusion of NAC for individual patients [44–46]. This forecasting technology leverages biologically-inspired mechanistic models that describe the key mechanisms driving the therapeutic response of the patient’s tumor to the prescribed NAC regimen by means of a set of partial differential equations [28, 30, 47]. The personalization of these mechanistic models can be achieved by adjusting their parameters for each individual patient. Towards this end, longitudinal anatomic and quantitative magnetic resonance imaging (MRI) data can provide patient-specific, spatiotemporally resolved information on tumor cell density, the local anatomy of the host tissue, and vasculature over the three-dimensional geometry of the affected breast [28, 30, 47]. Thus, if the model is personalized with MRI data acquired early in the course of NAC, ensuing computer simulations can provide a patient-specific forecast of therapeutic response at the conclusion of the prescribed NAC regimen and prior to surgery. Then, this tumor forecast would provide an early prediction of pCR status that could guide the treating oncologist in implementing potential treatment adjustments for individual patients. Indeed, patient-specific computational forecasts of breast cancer response to alternative NAC plans have shown that not all patients may benefit from the same NAC regimen and that these computational predictions may provide guidance in defining superior personalized treatment strategies [45, 48].

While anatomic and quantitative MRI data may provide rich spatially-resolved information about the architecture and physiology of the tumor and host tissue, there are two central limitations in the use of these data types for the patient-specific parameterization of spatiotemporal mechanistic models of tumor growth and therapeutic response: the scarcity of longitudinal MRI measurements in the clinical setting, and the inability to probe the tumor biology at cellular scale [29, 30, 47, 49]. The limited number of imaging datasets over time poses a central challenge for the patient-specific determination of key model parameters with a dynamic nature (e.g., tumor cell proliferation, mobility, drug-induced cytotoxic effects). Global variance-based sensitivity analysis [30, 50] provides a rigorous, systematic approach to identify the dominant model parameters that characterize the mechanisms driving tumor growth and response to treatment within a mechanistic model. These driving mechanisms require patient-specific parameterization, but the rest of the model parameters can be fixed to a constant value for all patients. Hence, global variance-based sensitivity analysis enables the definition of reduced models that approximate the overall tumor dynamics using the original model formulation, but only requiring the patient-specific identification of a reduced number of dominant parameters. Furthermore, the cytotoxic action of common chemotherapeutic agents is known to vary at cellular level [51, 52], ultimately depending on the specific genetic profile of the cancer cell subpopulations within a patient’s tumor and their evolution during NAC [19, 21, 52–54]. This heterogeneity in tumor biology can be systematically examined by leveraging high-throughput, time-resolved, automated microscopy assays. This technology can evaluate in vitro the response of multiple cancer cell cultures (e.g., from standardized lines, animal xenografts, or patient tissue samples) to numerous therapeutic regimens involving one or more drugs delivered with multiple dosages and schedules [55–58]. In this experimental setup, established mechanistic models are also leveraged to quantify the pharmacodynamic effect of each drug regimen on tumor dynamics (e.g., on tumor cell proliferation) [57, 59, 60]. However, the use of this wealth of in vitro data to parameterize in vivo models of tumor growth and treatment response remains an unresolved challenge due to three main issues: the unique intratumoral cellular heterogeneity of an individual patient’s tumor [52, 54, 61], local dynamic variations in the in vivo microenvironment of tumor cells directly affecting therapeutic response (e.g., perfusion, mechanical state) [24, 62–65], and the high number of parameters in the formulation of pharmacodynamic models [57, 59, 60].

Here, we propose a multiscale framework that integrates in vivo MRI data characterizing the morphology and function of the tumor and host tissue, in silico estimates of tumor dynamics during NAC, and in vitro measurements of TNBC cell response to NAC drug combinations to constrain and identify the driving mechanisms of organ-scale models of TNBC response to NAC. The in vivo and in silico data characterize breast cancer growth and treatment response at the organ scale, while the in vitro data provide information of NAC drug–drug interactions and their cytotoxic effects at the cellular scale. We propose to combine these multiscale, multimodal datasets to define the parameter space in which an organ-scale biomechanistic model of breast cancer growth and NAC response operates. Our multiscale framework further aims at determining a reduced set of model parameters that drive tumor dynamics according to the model formulation, and thus require patient-specific calibration. Hence, this reduced set of parameters could be calculated for each patient by exclusively using in vivo MRI data acquired early during the course of NAC to ultimately perform accurate predictions of therapeutic outcomes. To bring forth and analyze our multiscale framework, we also present a new organ-scale mechanistic model of breast cancer growth and response to NAC that couples the essential mechanisms underlying tumor dynamics with an explicit formulation of the prescribed NAC drugs’ pharmacokinetics and pharmacodynamics [30, 44, 45]. Given that NAC regimens usually consist of drug combinations, we adopt a recent pharmacodynamics model [57] that generalizes prior formulations of the pharmacodynamics of multidrug regimens and successfully describe the combined effects of multiple drug pairs over a range of tumor cell lines. The resulting organ-scale model features fifteen parameters that could be eligible for personalized parameterization with in vivo MRI data to perform tumor forecasting. To reduce this parameter set, we identify the driving parameters of our model using a global variance-based sensitivity analysis [30, 50] over a parameter space constructed by integrating in silico estimates informed by patient-specific in vivo MRI data and in vitro pharmacodynamic measurements. In particular, we experimentally constrain the parameters accounting for drug pharmacodynamics via time-resolved, high-throughput, automated microscopy assays that capture drug-induced changes in the proliferation rates of four TNBC cell lines (HCC1143, SUM149, MDAMB231, and MDAMB468). The resulting in vitro parameter ranges are then scaled to clinically-relevant values through computer simulations with our mechanistic model. In this work, we focus on two commonly used NAC regimens: doxorubicin plus cyclophosphamide, and paclitaxel plus carboplatin [3, 12, 45, 66]. Additionally, the sensitivity analysis is carried out in two representative scenarios of breast cancer in the clinical setting corresponding to a well-perfused and a poorly-perfused tumor, which are respectively extracted from in vivo MRI data from two TNBC patients. Furthermore, we show that computer simulations of the original and reduced model emanating from the sensitivity analysis can equally reproduce the clinical outcomes of NAC under the two NAC drug combination explored in this study, ranging from pCR to progressive disease. Thus, the present work has three main contributions: (i) a new organ-scale mechanisic model to describe TNBC response to NAC, (ii) a multiscale framework to constrain the parameter space of the model, and (iii) a global sensitivity analysis within this multiscale framework to identify a reduced set of parameters that suffice to capture the main dynamics of the model. As discussed in Sect. 4, future studies with the reduced model from this work will seek to investigate its predictive power in a diverse cohort of TNBC patients.

The remainder of this work is organized as follows. The next section describes the data used in this study, the mechanistic model of breast cancer growth and NAC response, the numerical methods for the spatiotemporal discretization of the model to enable computer simulations, and the formulation of the global variance-based sensitivity analysis. Then, we present the results of this sensitivity analysis along with a set of illustrative computer simulations of the model. Finally, we discuss the results and implications of our work, its limitations, and potential avenues of research in future studies.

## Methods

### MRI data

The MRI datasets for this study were obtained from a database of patient-specific, longitudinal anatomic and quantitative MRI in vivo measurements of breast cancer response to NAC at multiple time points before and during the course of the neoadjuvant regimen. This database has been presented in detail in Ref. [45], so here we present only the salient details and briefly summarize the corresponding preprocessing methods. The patient data were collected following an institutional review board-approved and HIPAA-compliant protocol, where patients provided informed consent to participate in a longitudinal MRI study throughout the course of their standard-of-care neoadjuvant therapy. For each breast cancer patient, five MRI data types were acquired at each scan session: (1) a pre-contrast T1 map, (2) a pre-contrast B1 field map to correct for radiofrequency inhomogeneity, (3) diffusion-weighted MRI (DW-MRI) data, (4) dynamic contrast-enhanced MRI (DCE-MRI) data, and (5) two high-resolution, T1-weighted anatomical scans (pre- and post-contrast). Then, the preprocessing pipeline of these MRI datasets consisted of four steps. First, an intra-scan rigid registration was applied to the MRI datasets collected within each scan session to correct for motion. Second, the tumor regions of interest (ROIs) were identified based on post-contrast scans using a fuzzy c-means algorithm [67]. Additionally, tissue properties related to perfusion-permeability of the vasculature were quantified by analyzing the DCE-MRI data with the Kety-Tofts model, which is a standard model describing the exchange of contrast agent between the plasma and tissue spaces [68]. The DW-MRI data were also analyzed to return maps of the apparent diffusion coefficient (ADC) of water. Third, an inter-scan registration was leveraged to align the images and calculated maps across all of the imaging sessions into a common geometric domain unique to each patient. This inter-scan registration relied on a non-rigid algorithm with a constraint that preserves the tumor volumes at each time point (see [45], although alternative non-rigid registration algorithms are available in the literature [69–72]). Finally, the last step of the preprocessing pipeline consisted of the calculation of the imaging-based quantities to be used within the spatiotemporal mechanistic model (see Sect. 2.3 and Refs. [30, 44, 45, 73–75]). In paticular, the tumor cell density maps were approximated from the voxel-based ADC values within the tumor ROI [30, 34, 44, 45, 76, 77], fibroglandular and adipose tissues were segmented based on enhancement in the DCE-MRI data by leveraging a k-means clustering algorithm, and a normalized perfusion map representing the spatial distribution of blood volume fraction within the breast tissue was calculated using the area under the dynamic curve (AUC) for each voxel in the DCE-MRI data [45]. The interested reader is referred to Supplementary Methods S1 and Ref. [45] for further information on MRI data acquisition and preprocessing.

**Fig. 1:**
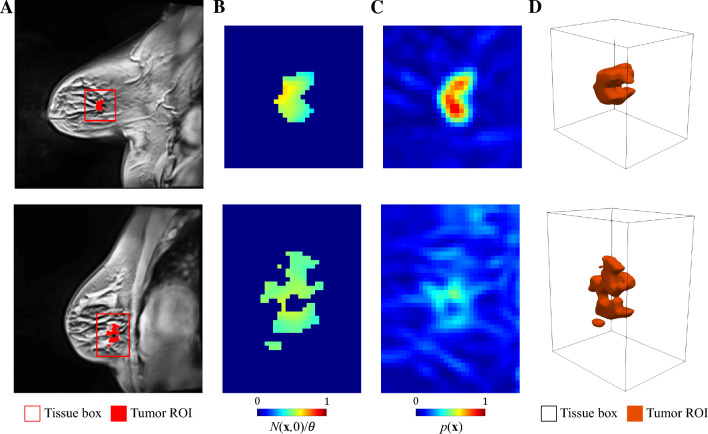
Patient-specific, MRI-informed tumor scenarios for the sensitivity analysis. Panels **A-D** show the in vivo MRI measurements characterizing the two TNBC cases considered in this study: a well-perfused tumor (top row) and a poorly perfused tumor (bottom row). Panel **A** presents a contrast-enhanced $T_1$-weighted sagittal image showing the patient’s breast anatomy and the tissue box containing the tumor region of interest (ROI) for each scenario. Panel **B** shows the corresponding tumor cell density maps over a sagittal section of the tissue box. These tumor cell density maps were calculated from ADC measurements obtained from DW-MRI data and normalized with respect to the tissue carrying capacity ($N(\textbf{x},t)/\theta$, see Sect. 2.3.1). Panel **C** depicts the local perfusion map over the same sagittal section of the tissue box for each tumor case. These perfusion maps were computed from DCE-MRI data ($p(\textbf{x})$, see Sect. 2.3.3). Finally, panel **D** shows a 3D rendering of each tumor ROI within the corresponding tissue box

The well-perfused and poorly-perfused breast tumor scenarios considered in the sensitivity analysis performed in this work were extracted from the MRI datasets collected for two TNBC patients from the aforementioned database (see Supplementary Methods S2 for further detail). In both cases, the TNBC was an invasive ductal carcinoma. While previous computational modeling and forecasting efforts of breast cancer response to NAC consider the tumor dynamics over the patient-specific anatomy of the affected breast [44, 45, 47, 73, 75–77], here we consider a box of breast tissue from each patient including their tumor as well as a surrounding region of healthy tissue. Figure 1 shows a 3D rendering and representative sagittal sections of the tissue box of the well-perfused and poorly-perfused breast tumor scenarios, along with their original location in the patient’s breast. The rationale for selecting this reduced geometry is to minimize the computational resources for the sensitivity analysis, which requires a large number of model simulations (see Sect. 2.5). In particular, we estimated that the box size for each scenario was sufficient to accommodate all potential outcomes of NAC for the varying parameter combinations used within the sensitivity analysis (i.e., ranging from tumor elimination to consistent growth at the end of treatment), while also limiting the computational resources required to perform a model simulation for each parameter combination.

### High-throughput, automated microscopy data

Four TNBC cell lines representing different TNBC subtypes [78] were used in this study. Each cell line was engineered to express histone 2B-monomeric red fluorescent protein 1 (H2BmRFP1) using recombinant lentiviruses as described previously [79]: HCC-1143, MDA-MB-231, MDA-MB-468, and SUM-149. While HCC-1143 and SUM-149 cell lines were derived from a patient with ductal carcinoma, MDA-MB-231 and MDA-MB-468 were derived from metastatic sites of patients with breast adenocarcinoma. The TNBC subtypes [78] that the cell lines represent are: basal-like 1 (HCC-1143, MDA-MB-468), basal-like 2 (SUM-149), and mesenchymal (MDA-MB-231). Sum-149 cells were cultured in Ham’s F-12 medium containing 5% fetal bovine serum, 1 $\upmu$g/ml hydrocortisone and 5 $\upmu$g/ml insulin. All other cell lines were cultured in Dulbecco’s Modified Eagle’s Medium (DMEM) containing 10% fetal bovine serum. Doxorubicin HCl (Pfizer, NDC 0069-3032-20) was obtained from the Vanderbilt Clinic, while 4-hydroperoxy cyclophosphamide (also known as perfosfamide; the active metabolite form of the prodrug cyclophosphamide), carboplatin, and paclitaxel were purchased from MedChemExpress (cat# HY-117433, HY-17393, and HY-B0015, respectively). Drug combination studies were performed in the Vanderbilt High Throughput Screening Facility as previously described [57]. Briefly, cells were seeded at approximately 400 cells per well in 384-well plates and allowed to adhere overnight. A preliminary image of each plate was taken approximately 8 h after seeding to verify sufficient cells for each experiment. Fluorescence microscopy images were taken on an ImageXpress Micro XL (Molecular Devices). Medium containing drugs and 5 nM Sytox Green (to detect dead cells) was added and replaced after 72 h. Cells were imaged over approximately 120 h of drug exposure. Cell counts were determined using custom-written image segmentation software developed in Python using scikit-image [80] and run in parallel using RabbitMQ/Celery. Changes in viable cell count over time were used to extract drug-induced proliferation (DIP) rates as previously described [81, 82]. Finally, DIP rate values for each drug–drug or single agent drug condition were assessed for any synergistic activity using the multi-dimensional synergy of combinations (MuSyC) formalism [57]. Supplementary Tables S1 and S2 summarize the distribution of experimental measurements of the MuSyC pharmacodynamic parameters in the four TNBC cell lines treated with doxorubicin plus cyclophosphamide and paclitaxel plus carboplatin, respectively.

### Mechanistic model of breast cancer dynamics and NAC response

**Fig. 2:**
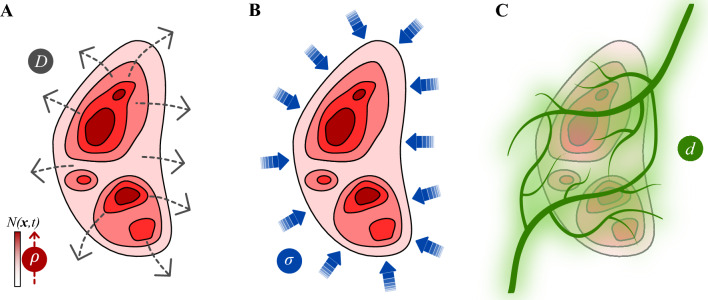
Mechanistic modeling of breast cancer growth and response to NAC. Panel **A** illustrates the baseline tumor dynamics in terms of tumor cell density $N(\textbf{x},t)$ as a combination of mobility and net proliferation, as shown in Eq. (1). We model tumor cell mobility with a diffusion operator that is characterized by a tumor cell diffusion coefficient *D*. This formulation aims to capture the expansion of the tumor over the surrounding host tissue. The net proliferation of the tumor cells is formulated with a logistic term, which is driven by the net proliferation rate $\rho$ and locally increases the tumor cell density over time. Panel **B** shows that, as solid tumors deform the host tissue in which they are developing, this process induces mechanical stress fields $\sigma$ that are known to inhibit tumor growth. In our model, we constrain tumor cell mobility with the local mechanical stress field via Eq. (2). Panel **C** shows that the NAC drugs considered in this study reach the tumor through the bloodstream after being delivered through intravenous injection. Since these drugs have a cytotoxic action on tumor dynamics (i.e., they induce tumor cell death), Eq. (8) locally modifies the tumor cell net proliferation according to the joint effect of the local concentration *d* of each drug pair during the course of NAC. In particular, our model couples the pharmacokinetics and pharmacodynamics of the drugs in each NAC regimen via Eqs. (8)–(14), also accounting for drug synergy interactions according to the MuSyC framework (see Fig. 3). Panel **C** was created using BioRender

Our mechanistic modeling framework relies on a biologically inspired approach that aims at describing the main spatiotemporal mechanisms underlying tumor response to NAC at organ scale, such that the driving phenomena in the model can be parameterized for each individual patient by leveraging longitudinal in vivo MRI measurements collected before and during the course of NAC [28, 30, 44, 45, 47]. In particular, we couple a previous mechanically constrained mechanistic model of breast cancer response to NAC [73–75] with a pharmacodynamics model that explicitly captures the combined effect on tumor cell proliferation of drug pairs in NAC regimens [57, 60], while also accounting for drug pharmacokinetics [44–47]. Figure 2 illustrates the mechanisms in the model, which we now describe in detail.

#### Tumor dynamics

Let $\textbf{x}$ denote the position vector within the spatial domain $\Omega$, which consists of a box of breast tissue including the tumor (see Sect. 2.1), and let *t* denote time within the interval [0, *T*], such that *T* represents the time horizon when model outcomes will be assessed (e.g., after the completion of NAC). We model breast cancer dynamics in terms of tumor cell density $N(\textbf{x},t)$ as

1 $$\begin{aligned} \frac{\partial N}{\partial t} = \nabla \cdot \left( D\nabla N \right) + \rho N \left( 1 - \frac{N}{\theta } \right) {.} \end{aligned}$$

The right-hand-side of Eq. (1) describes the dynamics of $N(\textbf{x},t)$ as a combination of tumor cell mobility and net proliferation by leveraging a diffusion operator and a logistic term, respectively. In this equation, $D(\textbf{x},t)$ denotes the tumor cell diffusivity, which quantifies tumor cell mobility. Additionally, $\rho (\textbf{x},t)$ represents the tumor cell net proliferation rate, which encompasses the baseline balance of tumor cell proliferation and death as well as the therapeutically-induced tumor cell death due to the cytotoxic action of the prescribed drugs. Hence, a positive value of $\rho (\textbf{x},t)$ contributes to tumor growth, whereas a negative value leads towards tumor elimination. Finally, $\theta$ denotes the breast tissue carrying capacity, which represents the maximum admissible tumor cell density at any point $\textbf{x}\in \Omega$. The reaction-diffusion modeling paradigm in Eq. (1) has been successfully employed to represent and forecast the dynamics of breast cancer growth and response to NAC of individual patients [44, 45, 73, 74, 83], as well as the development and treatment response of other tumors [31–37, 84, 85]. Following this modeling paradigm, Eq. (1) is coupled with the zero-flux boundary condition $\nabla N \cdot \textbf{n} = 0$ on $\Gamma =\partial \Omega$, where $\textbf{n}$ is an outward unit vector normal to the boundary of the tissue box (see Sect. 2.1). Hence, this boundary condition is consistent with the assumption that the tissue box suffices to spatially hold the diverse spectrum of the dynamics of breast cancer response to be investigated during the sensitivity analysis in each tumor scenario.

#### Mechanical coupling

Tumor growth induces the deformation and accumulation of mechanical stress in the host tissue [62, 63, 86, 87]. This phenomenon is usually termed the tumor mass effect. Since mechanical stress is known to exert an inhibitory effect on tumor growth [62, 63, 86, 87], we constrain the tumor cell diffusion coefficient $D(\textbf{x},t)$ in Eq. (1) with the local tumor-induced mechanical stress field in the breast:

2 $$\begin{aligned} D(\textbf{x},t) = D_0 e^{-\gamma _N\sigma _v(\textbf{x},t)}. \end{aligned}$$

In Eq. (2), $D_0$ represents the tumor cell diffusivity in the absence of mechanical inhibition, $\gamma _N$ denotes an empirical coupling constant, and $\sigma _v(\textbf{x},t)$ is the von Mises stress, which is a scalar stress metric calculated as

3 $$\begin{aligned} \sigma _v{} & {} = \left( \sigma ^2_{11}+\sigma ^2_{22}+\sigma ^2_{33} - \sigma _{11}\sigma _{22} - \sigma _{22}\sigma _{33} -\sigma _{11}\sigma _{33} \right. \nonumber \\{} & {} \quad \left. + 3 \left( \sigma ^2_{12} + \sigma ^2_{23} + \sigma ^2_{13} \right) \right) ^{1/2}, \end{aligned}$$

where $\sigma _{ij}$ (with $i,j=1,2,3$) are the components of the second-order mechanical stress tensor $\varvec{\sigma }(\textbf{x},t)$. This mechanically coupled approach has been shown to render superior predictions of breast tumor dynamics during NAC with respect to a baseline model without mechanics [73, 74], and it has also been adopted in patient-specific, mechanically constrained models of brain and prostate cancer [34, 88, 89].

To calculate the mechanical stress tensor $\varvec{\sigma }(\textbf{x},t)$, we assume that the tumor-induced mechanical deformation of breast tissue is sufficiently slow to disregard inertial effects and that, hence, it is governed by quasistatic equilibrium [29, 34, 37, 73–75, 88, 89], which is modeled as

4 $$\begin{aligned} {\mathbf{\nabla} }\cdot {\mathbf{\sigma} } = \textbf{0}. \end{aligned}$$

Additionally, we set Winkler-inspired boundary conditions $\mathbf {\sigma n} = -k_{\text {w}}\textbf{u}$ in $\Gamma =\partial \Omega$, where $\textbf{u}$ is the displacement vector and $\textbf{n}$ is an outward unit vector normal to the tissue box boundary (see Sect. 2.1). These boundary conditions aim at modeling the mechanical constraint to tumor growth imposed by the tissues surrounding the box domain in each tumor scenario [88, 89]. The parameter $k_ {w}$ in Eq. (4) quantifies the mechanical stresses induced by the neighboring tissues per unit of the boundary displacement.

Linear elasticity has been widely used to describe the tumor-induced deformation of the host tissue in clinically oriented models of tumor growth and treatment response [29, 34, 37, 73–75, 88, 89]. Additionally, the breast is a histologically heterogeneous organ composed of fibroglandular and adipose tissue, with the former type of tissue exhibiting a higher elastic modulus [73–75]. Hence, breast tissue can be modeled as a linear elastic, heterogeneous, isotropic material with a constitutive equation given by

5 $$\begin{aligned} {\sigma } = \lambda \left( {\nabla }\cdot \textbf{u} \right) \textbf{I} + \mu \left( {\nabla \textbf{u}} + {\nabla \textbf{u}}^T \right) - g_N \frac{N}{\theta } \textbf{I}, \end{aligned}$$

where $\varvec{\sigma }(\textbf{x},t)$ is the mechanical stress tensor, $\textbf{u}(\textbf{x},t)$ is the displacement vector, $\mathbf {\nabla }^s\textbf{u}$ is the symmetric gradient of the displacement field (i.e., the strain tensor in linear elasticity, $\varvec{\varepsilon }=\mathbf {\nabla }^s\textbf{u}=\left( \mathbf {\nabla u} + \mathbf {\nabla u}^T \right) /2$), $\textbf{I}$ is the second-order identity tensor, and $\lambda (\textbf{x})$ and $\mu (\textbf{x})$ are the local Lamé coefficients. These mechanical parameters are related to the local tissue Young’s elastic modulus *E* and Poisson’s ratio $\nu$ as $\lambda = {E\nu }/{((1+\nu )(1-2\nu ))}$ and $\mu = {E}/{(2(1+\nu ))}$. Thus, in Eq. (5), the first two terms in the right-hand side correspond to the usual linear elasticity constitutive equation while the third term describes the tumor mass effect as a growth-induced phenomenon, which is characterized by the tumor-induced solid stress $g_N$ and is proportional to the normalized tumor cell density $N(\textbf{x},t)/\theta$ [29, 34, 37, 73–75, 88, 89].

#### Pharmacodynamics and pharmacokinetics of NAC drugs

*2.3.3.1. Drug pharmacodynamics*

Standard NAC drug combinations induce tumor cell death, which ultimately reduces or even eliminates the patient’s tumor burden [2, 3, 7–9]. The effect of NAC drugs on tumor cells is usually quantified in vitro with concentration-dependent pharmacodynamic models, which can further accommodate synergistic effects for multidrug regimens (see Sect. 2.2) [57, 59, 60]. However, the relatively large number of parameters in these pharmacodynamic models usually precludes their use in organ-scale clinically-oriented models because the patient-specific clinical and imaging data available for their determination is insufficient. The standard approach to account for the effect of chemotherapeutic drugs in mechanistic models of tumor growth and treatment response in the clinical setting consists of introducing a term to counteract the proliferation mechanism in the baseline model. For example, some models extend the equation governing tumor dynamics (e.g., Eq. (1)) with a reaction term proportional to the tumor burden and the drug concentration, while others opt to directly adjust the net proliferation rate with a similar concentration-dependent term after the delivery of each drug dose [44, 45, 56, 58, 90–94].

Here, we propose to explicitly couple a full pharmacodynamic model with the net proliferation rate $\rho (\textbf{x},t)$ in Eq. (1). Our underlying assumptions are: (1) the same pharmacodynamic paradigm can be used to describe the response of TNBC cells in vitro and in vivo, and (2) the pharmacodynamic parameters of each NAC drug pair are either in the same range for both in vitro and in vivo scenarios or can be scaled accordingly through computer simulations of our mechanistic model (see Sect. 2.3.4). It is important to note that this last assumption does not mean that we determine the pharmacodynamic parameters for a given NAC drug combination in vitro and then directly use these values within the definition of $\rho (\textbf{x},t)$ in Eq. (1) to predict the response of any patient’s tumor to that NAC regimen. Rather, we define the value ranges for the pharmacodynamic parameters using the in vitro data in Sect. 2.2 and, then, use those ranges to construct the parameter space to constrain our mechanistic model for sensitivity analysis in the clinical setting (see Sect. 2.3.4 below). In the next section, we describe the formulation of the drug-informed net proliferation rate, which relies on the normalization of drug concentrations and drug effects.

*2.3.3.2. Normalization of drug pharmacodynamics*

We adjust the proliferation rate $\rho (\textbf{x},t)$ in Eq. (1) using a normalized version of the MuSyC model [57] informed by drug pharmacokinetics. This choice of the MuSyC model is motivated by its use to measure the combined drug effects and drug–drug synergies in the experiments with the four TNBC cell lines (see Sect. 2.2). Hence, we can directly use the experimental measurements of the pharmacodynamic parameters in the MuSyC equation to estimate value ranges for the same parameters in the organ-scale simulated scenarios considered in this work (see Sect. 2.3.4 below). Indeed, the normalization approach serves two purposes to facilitate the use of the MuSyC model in both in vitro and in vivo scenarios. First, while drug concentrations can be precisely controlled in experimental settings, the corresponding values within the tumor and host tissue of an individual patient cannot be measured with routine clinical imaging methods. However, our mechanistic model does not necessarily require the precise value of drug concentrations, but rather their relative temporal change following the delivery of each dose (e.g., due to tumor consumption and elimination) [44, 45, 56, 58, 90–94]. Second, the maximal drug effects may differ between an in vitro setting and a patient-specific in vivo scenario. For example, standard NAC drugs for TNBC have been observed to reduce the net proliferation rate of breast tumor cell populations and limit their growth [51, 56, 58, 95]. In particular, Supplementary Tables S1 and S2 shows that the maximal drug effects measured in the in vitro experiments for this work resulted in either a reduction of the tumor cell net proliferation or the induction of mild tumor cell net death (i.e., a negative value of the drug-induced tumor cell net proliferation rate). However, the delivery of the same drugs in routine NAC regimens is known to achieve the complete tumor elimination in some breast cancer patients (i.e., a pCR) [2, 3, 7–9]. As for drug concentrations, from a mechanistic modeling perspective it suffices to quantify the drug-induced relative change in tumor cell proliferation to capture the chemotherapeutic effect of a given drug regimen on tumor dynamics in both preclinical and clinical scenarios [44, 45, 56, 58, 90–94].

To construct the normalized MuSyC model, let us first define $\rho _0$ as the global baseline value of the tumor cell net proliferation before the onset of NAC, and $C_i$ ($i=1,2$) as the half-maximal effective concentration of each NAC drug in a particular regimen (i.e., the drug concentration producing half of the maximal effect for each individual drug). Then, our approach relies on normalizing the drug concentrations $d_i(\textbf{x},t)$ with respect to their corresponding $C_i$ and normalizing the maximal drug effects on tumor cell proliferation $E_i$ with respect to $\rho _0$. Thus, we calculate the normalized drug concentrations ${\hat{d}}_i$ and the normalized maximal drug effects ${\hat{E}}_i$ on tumor cell net proliferation as

6 $$\begin{aligned} {{\hat{d}}_i(\textbf{x},t)=d_i(\textbf{x},t)/C_i} \end{aligned}$$

and

7 $$\begin{aligned} {{\hat{E}}_i=E_i/\rho _0,} \end{aligned}$$

respectively. These parameters are defined for each of the two drugs ($i=1,2$) used in the NAC regimens considered in this work (i.e., doxorubicin plus cyclophosphamide, and paclitaxel plus carboplatin). Using the previous definitions within the MuSyC equation provided in Ref. [57], we express the tumor cell net proliferation rate $\rho (\textbf{x},t)$ from Eq. (1) in terms of the normalized drug concentrations ${\hat{d}}_1(\textbf{x},t)$ and ${\hat{d}}_2(\textbf{x},t)$ in each NAC drug regimen as

8 $$\begin{aligned} \rho = \rho _0 \frac{ g_0\left( {\hat{d}}_1,{\hat{d}}_2\right) + g_1\left( {\hat{d}}_1,{\hat{d}}_2\right) {\hat{d}}_1^{h_1}{\hat{E}}_1 + g_2\left( {\hat{d}}_1,{\hat{d}}_2\right) {\hat{d}}_2^{h_2}{\hat{E}}_2 + g_3\left( {\hat{d}}_1,{\hat{d}}_2\right) {\hat{d}}_1^{h_1}{\hat{d}}_2^{h_2}{\hat{E}}_3}{ g_0\left( {\hat{d}}_1,{\hat{d}}_2\right) + g_1\left( {\hat{d}}_1,{\hat{d}}_2\right) {\hat{d}}_1^{h_1} + g_2\left( {\hat{d}}_1,{\hat{d}}_2\right) {\hat{d}}_2^{h_2} + g_3\left( {\hat{d}}_1,{\hat{d}}_2\right) {\hat{d}}_1^{h_1}{\hat{d}}_2^{h_2} }, \end{aligned}$$

where

9 $$\begin{aligned} g_0\left( {\hat{d}}_1,{\hat{d}}_2\right){} & {} = C_1^{h_1} + C_2^{h_2} + \left( C_1\alpha _2{\hat{d}}_1\right) ^{h_1} + \left( C_2\alpha _1{\hat{d}}_2\right) ^{h_2} {,} \end{aligned}$$

10 $$\begin{aligned} g_1\left( {\hat{d}}_1,{\hat{d}}_2\right){} & {} = C_1^{h_1} + C_2^{h_2} + \left( C_1\alpha _2{\hat{d}}_1\right) ^{h_1} + \alpha _2^{h_1}\left( C_2{\hat{d}}_2\right) ^{h_2} {,} \end{aligned}$$

11 $$\begin{aligned} g_2\left( {\hat{d}}_1,{\hat{d}}_2\right){} & {} = C_1^{h_1} + C_2^{h_2} + \alpha _1^{h_2}\left( C_1{\hat{d}}_1\right) ^{h_1} + \left( C_2\alpha _1{\hat{d}}_2\right) ^{h_2} {,} \end{aligned}$$

12 $$\begin{aligned} g_3\left( {\hat{d}}_1,{\hat{d}}_2\right){} & {} = \left( C_1\alpha _2\right) ^{h_1} + \left( C_2\alpha _1\right) ^{h_2} + \alpha _1^{h_2}\left( C_1\alpha _2{\hat{d}}_1\right) ^{h_1} \nonumber \\{} & {} \quad + \alpha _2^{h_1}\left( C_2\alpha _1{\hat{d}}_2\right) ^{h_2} {,} \end{aligned}$$

13 $$\begin{aligned} {\hat{E}}_3{} & {} = \left( 1+\beta \right) \min \left( {\hat{E}}_1,{\hat{E}}_2\right) - \beta {.} \end{aligned}$$

**Fig. 3:**
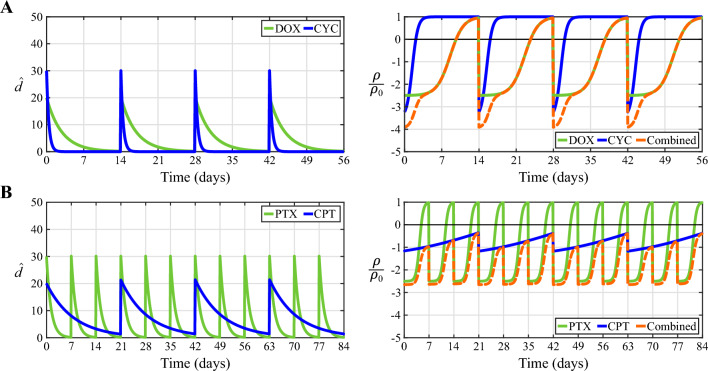
Pharmacokinetics and pharmacodynamics of the NAC drug pairs. We consider two NAC regimens: four 2-week cycles of doxorubicin plus cyclophosphamide (DOX and CYC, panel **A**), and four 3-week cycles of weekly paclitaxel plus 3-weekly carboplatin (PTX and CPT, panel **B**). In each panel, the left plot illustrates the pharmacokinetics of either drug in the regimen at an arbitrary spatial point of perfused tissue $\textbf{x}\in \Omega$ (i.e., the temporal change in local drug concentration according to Eq. (14) in Sect. 2.3.3). The peaks in the normalized drug concentration ${\hat{d}}$ for each drug correspond to the days of delivery. The right plot in each panel shows the corresponding drug-induced changes in tumor cell proliferation caused by each drug alone as well as in combination. In the latter case, synergistic effects emerge according to the MuSyC framework [57]. The drug-induced changes are calculated by combining the pharmacodynamics and pharmacokinetics formulations within our proposed model of breast cancer response to NAC (i.e., Eqs. (8) and (14) in Sect. 2.3.3)

Notice that the numerator and denominator of Eq. (8) reflect the original formulation of the MuSyC model as proposed in Ref. [57]. The only change that we introduce is the normalization of the drug concentration and maximal effects according to Eqs. (6)–(7) in the terms of the numerator and denominator. In Eqs. (8)–(13), $h_i$ and $\alpha _i$ ($i=1,2$) respectively denote the Hill coefficient and the synergy of potency of each drug in the regimen, which measures the increase in potency for a given concentration when the drugs are delivered in combination. The parameter $\beta$ is the synergy of efficacy, which defines the additional effect $E_3$ on tumor cell proliferation achieved when the two drugs in the NAC regimen are given concomitantly, as indicated in Eq. (13). Thus, in our modeling framework, the parameter set comprised of $h_1$, $h_2$, ${\hat{E}}_1$, ${\hat{E}}_2$, $C_1$, $C_2$, $\alpha _1$, $\alpha _2$, and $\beta$ characterizes the pharmacodynamics of an NAC drug combination. Figure 3 provides an example to illustrate the pharmacodynamics of the two NAC regimens considered in this study.

*2.3.3.3. Drug pharmacokinetics*

Following the delivery of each drug dose, pharmacokinetic models describe the temporal dynamics of the drug concentration in the patient’s body (e.g., drug infusion, systemic distribution, consumption, and elimination) [96–101]. These pharmacokinetic models result in a terminal decaying phase that dominates after a few hours or days of drug delivery [99, 101–106]. Thus, an exponentially decaying formulation provides a reasonable approximation of the relevant drug pharmacokinetics during the time scale of chemotherapeutic regimens, since each drug dose is usually delivered every 1–3 weeks. Indeed, this modeling strategy is a common approach to approximate drug pharmacokinetics in mechanistic models of tumor response to systemic therapies [44, 45, 48, 90–92]. Here, we adopt the exponential decay formulation for drug pharmacokinetics and we further extend it to accommodate the drug distribution across the tumor and breast tissue, as proposed in [44, 45].

*2.3.3.4. Normalization of drug pharmacokinetics*

We define the normalized drug concentrations ${\hat{d}}_i (\textbf{x},t)$ in Eqs. (8)–(12) as

14 $$\begin{aligned} {\hat{d}}_i (\textbf{x},t) = \sum _{j=1}^{n_i} {\hat{d}}_{m,i} p(\textbf{x})e^{-\gamma _i\left( t-t_{ij}\right) }H(t-t_{ij}){,} \end{aligned}$$

where subindices *i* and *j* denote each drug in the NAC regimen ($i=1,2$) and the number of each dose in the treatment plan of the *i*-th drug ($j=1,\ldots ,n_i$), respectively. Hence, $t_{ij}$ represents the delivery time of the *j*-th dose of the *i*-th drug, which only contributes to the sum in Eq. (14) for times $t>t_{ij}$ due to the Heaviside function $H(t-t_{ij})$. The parameter ${\hat{d}}_{m,i}$ ($i=1,2$) is the normalized maximum drug concentration, which is calculated as

15 $$\begin{aligned} {{\hat{d}}_{m,i}=d_{m,i}/C_i,} \end{aligned}$$

where $d_{m,i}$ is the nominal maximal drug concentration and $C_i$ is the half-maximal effective concentration of the *i*th drug (see Sect. 2.3.3.2 above). The spatial map $p(\textbf{x})$ describes blood perfusion in the tumor and the breast tissues, and it is calculated from patient-specific DCE-MRI data (see Sect. 2.1). Additionally, the parameter $\gamma _i$ ($i=1,2$) is the decay rate of each drug in the NAC regimen. Figure 3 depicts an example of the pharmacokinetics of the drugs participating in the two NAC regimens considered in this study.

#### Parameter values and ranges

The global variance-based sensitivity analysis presented in this work includes a total of $n_{p}=15$ parameters of our mechanistic model, which were selected based on our previous studies on patient-specific tumor forecasting and drug pharmacodynamic modeling [44, 45, 47, 57, 73–75]. These parameters are: the baseline tumor cell diffusivity ($D_0$), the baseline tumor cell net proliferation ($\rho _0$), the drug Hill coefficients ($h_1,h_2$), the half-maximal effective drug concentrations ($C_1,C_2$), the normalized maximal drug effects ($E_1,E_2$), the coefficients of drug synergy of potency ($\alpha _1,\alpha _2$), the coefficient of drug synergy of efficacy ($\beta$), the normalized maximal drug concentrations (${\hat{d}}_{m,1},{\hat{d}}_{m,2}$), and the drug decay rates ($\gamma _1,\gamma _2$). Table 1 shows the admissible ranges of each of these parameters in the sensitivity analysis for each NAC regimen. As observed in Table 1, the admissible values for some of the parameters participating in the sensitivity analysis spanned several orders of magnitude. In those cases, the parameters were sampled logarithmically with a uniform distribution. We also imposed this sampling technique for the majority of the pharmacodynamics parameters based on the experimentally-measured distributions of these parameters observed in previous works with the MuSyC model (see, e.g., Ref. [57]). The only exceptions are the normalized maximal drug effects ($E_1,E_2$) and the coefficients of drug synergy of efficacy ($\beta$). The rationale for the regular uniform sampling is that they can take both positive and negative values, and that values close to zero below the minimal order of magnitude in the range bounds reported in Table 1 resulted in a negligible effect of the parameter in our preliminary simulations of the model. For the regularly sampled parameters, the distribution of admissible values was assumed to be uniform within the reported bounds in Table 1. In the following, we describe how these parameter ranges were defined.

The admissible values for the baseline tumor cell diffusivity $D_0$ and net proliferation rate $\rho _0$ were set according to our previous patient-specific, imaging-informed modeling work on breast cancer response to NAC [44, 45, 47, 73–75]. The intervals of admissible values for the normalized maximum concentration ${\hat{d}}_{m,i}$ and the decay rate $\gamma _i$ of the drugs in each NAC regimen ($i=1,2$) were also constructed from these studies [44, 45, 47] as well as from values reported in the literature [51, 95, 98–106].

For each drug combination in the NAC regimens, the ranges for the Hill coefficients $h_i$, the half-maximal drug concentrations $C_i$, the synergy of potency coefficients $\alpha _i$ ($i=1,2$), and the synergy of efficacy coefficient $\beta$ were directly obtained from the in vitro measurements described in Sect. 2.2. This choice follows from the two assumptions underlying our pharmacodynamics model (see Sect. 2.3.3.1). Supplementary Tables S1 and S2 provide the average and 95% confidence interval for the values of these parameters for each of the four TNBC cell lines used in this study, which were obtained by fitting the MuSyC model to the corresponding experimental measurements (see Sect. 2.2). To construct the range of each of these parameters reported in Table 1, we took the lowest and highest value from the corresponding 95% confidence intervals across the four TNBC cell lines. Then, we rounded them to the nearest first decimal figure, such that the range length was always increased. Hence, these experimentally-sourced ranges in Table 1 were defined broadly to include all of the 95$\%$ confidence intervals obtained for each parameter as a result of fitting the MuSyC equation to the in vitro data collected for each TNBC line.

The experimentally-determined values of the normalized maximal drug effects ${\hat{E}}_i$ ($i=1,2$) for each NAC regimen only represented a reduction of tumor cell net proliferation or the induction of mild tumor cell death in each TNBC cell line (see Supplementary Tables S1 and S2). Consequently, the direct use of these values within our organ-scale mechanistic model resulted in simulations mainly exhibiting stable or progressive TNBC at the end of NAC. Thus, these experimentally determined values of ${\hat{E}}_i$ hinder the representation of all clinical NAC outcomes, ranging from tumor eradication (i.e., pCR) to progressive disease at NAC conclusion. To avoid this issue, we computationally estimated the corresponding clinically relevant ranges for ${\hat{E}}_i$ ($i=1,2$) in each drug regimen. To this end, we first ran a series of preliminary computer simulations with our model equipped with fixed values for the rest of the parameters within the ranges in Table 1, and varying the ${\hat{E}}_i$ for each drug in each regimen in an independent manner. The values of the fixed parameters were sampled initially around the median according to Table 1, and then within each quartile. The highest value of ${\hat{E}}_i$ was taken as ${\hat{E}}_i=0.5$ for all drugs because it provided an upper bound that was sufficiently high to ensure the simulation of progressive disease with our organ-scale mechanistic model. Then, these preliminary simulations suggested that setting ${\hat{E}}_{i,\min }=-5$ for both doxorubicin and cyclophosphamide and ${\hat{E}}_{i,\min }=-3$ for both paclitaxel and carboplatin sufficed to represent the diverse spectrum of clinical NAC outcomes from pCR to progressive disease (i.e., lower ${\hat{E}}_{i}$ values primarily led to total or nearly complete tumor eradication). We further confirmed this diversity of clinical outcomes by running the simulations corresponding to the two baseline samples of 1000 random parameter combinations leveraged to bring forth the global variance-based sensitivity analysis for each NAC regimen (see Sect. 2.5 as well as Figs. 5 and 9 in the Sect. 3). Finally, notice that the computational estimation of ${\hat{E}}_i$ is compatible with the two assumptions of our pharmacodynamics modeling framework in Sect. 2.3.3.1 (in particular, that some pharmacodynamics parameters can be scaled to organ-scale in vivo scenarios).

The rest of the model parameters that were not included in the sensitivity analysis were fixed according to literature values and our prior patient-specific, imaging-informed modeling efforts [34, 44, 45, 47, 73–75, 88, 89]. We set the tumor cell carrying capacity $\theta =2.02\cdot 10^6$ cells/$mm^{3}$, the mechanical coupling coefficient $\gamma _N=2.5$ 1/KPa, the Winkler boundary constant $k_{\text {w}}=0.20$ KPa/mm, the tumor-induced pressure $g_N=5$ KPa, the Poisson’s ratio $\nu =0.45$, and the Young’s modulus $E=3$ KPa. While our model can accommodate heterogeneous mechanical properties that can be spatially defined via MRI data [34, 44, 45, 47, 73–75, 88, 89], we opted for an average value over the breast tissue in an attempt to simplify and generalize the results of the sensitivity analysis beyond the particular background tissue architecture of the tumor scenarios considered in this study.

**Table 1:** List of parameters considered in the sensitivity analysis and corresponding value ranges

Parameter	Notation	Units	Sampling	NAC regimen	
				DOX and CYC	PTX and CPT
Baseline tumor cell diffusivity	$D_0$	$mm^{2}$/day	Logarithmic	$[1.0\cdot 10^{-6}, 1.0\cdot 10^{-1}]$	$[1.0\cdot 10^{-6}, 1.0\cdot 10^{-1}]$
Baseline tumor cell net proliferation	$\rho _0$	1/day	Logarithmic	$[1.0\cdot 10^{-3}, 1.0\cdot 10^{-1}]$	$[1.0\cdot 10^{-3}, 1.0\cdot 10^{-1}]$
Drug Hill coefficients	$h_1$	–	Logarithmic	[1.0, 2.5$\cdot 10^1$]	[1.0, 6.3]
	$h_2$	–	Logarithmic	[0.4, 3.2]	[0.3, 3.2]
Half-maximal effective drug concentrations	$C_1$	$\upmu$M	Logarithmic	[6.3$\cdot 10^{-4}$, 1.6$\cdot 10^{-2}$]	[$1.0\cdot 10^{-3}, 4.0\cdot 10^{-3}$]
	$C_2$	$\upmu$M	Logarithmic	[1.3$\cdot 10^{-1}$, 1.6$\cdot 10^{3}$]	[6.3$\cdot 10^{-2}$, 4.0$\cdot 10^{1}$]
Normalized maximal drug effects	${\hat{E}}_{1}$	–	Regular	[− 5, 0.5]	[− 3, 0.5]
	${\hat{E}}_{2}$	–	Regular	[-5, 0.5]	[− 3, 0.5]
Coefficients of drug synergy of potency	$\alpha _{1}$	–	Logarithmic	[$1.0\cdot 10^{-4}$, 1.3$\cdot 10^{3}$]	[$1.0\cdot 10^{-4}$, $1.0 \cdot 10^4$]
	$\alpha _{2}$	–	Logarithmic	[$1.0\cdot 10^{-4}$, 4.0$\cdot 10^{1}$]	[$1.0\cdot 10^{-4}$, 2.5]
Coefficient of drug synergy of efficacy	$\beta$	–	Regular	[$-$0.2, 0.3]	[$-$0.1, 0.2]
Normalized maximal drug concentrations	${\hat{d}}_{m,1}$	–	Logarithmic	[5.0, $1.0\cdot 10^3$]	[5.0, $1.0\cdot 10^3$]
	${\hat{d}}_{m,2}$	–	Logarithmic	[5.0, $1.0\cdot 10^3$]	[5.0, $1.0\cdot 10^3$]
Drug decay rates	$\gamma _1$	1/day	Regular	[0.3, 0.6]	[0.3, 1.1]
	$\gamma _2$	1/day	Regular	[1.7, 5.4]	[0.1, 0.2]

For each drug pair in the NAC regimens, parameters with subindex 1 correspond to the first drug (i.e., doxorubicin and paclitaxel) and parameters with subindex 2 correspond the second drug (i.e., cyclophosphamide and carboplatin)

*DOX* doxorubicin, *CYC* cyclophosphamide, *PTX* paclitaxel, *CPT* carboplatin

### Numerical methods

#### Spatial discretization

We discretize in space using Isogeometric Analysis (IGA), which is a recent generalization of the classic Finite Element Method [30, 107]. In particular, our spatial discretization relies on a standard isogeometric Bubnov–Galerkin approach using a three-dimensional $C^1$ quadratic B-spline space [30, 89, 90, 107]. The isogeometric meshes for the well-perfused and poorly-perfused tumor scenarios were constructed by upsampling the original voxel grid of the corresponding tissue boxes (see Fig. 1 and Sect. 2.1) for numerical accuracy (see Supplementary Methods S3 for further detail).

#### Time discretization

We partition the time domain [0, *T*] using a constant time step of 0.25 days [44, 75]. The initial time $t=0$ corresponds to the onset of NAC. We consider a standard doxorubicin plus cyclophosphamide regimen consisting of four 2-week cycles in which both drugs are delivered the first day of each cycle [3, 44, 45, 66] (see Fig. 3). Additionally, we consider a paclitaxel plus carboplatin regimen consisting of four 3-week cycles in which paclitaxel is administered weekly, while carboplatin is delivered on the first day of each cycle [44, 45, 66] (see Fig. 3). For both regimens, we set the time horizon $t=T$ at the end of the last cycle, such that $T=56$ days for the doxorubicin plus cyclophosphamide regimen and $T=84$ days for the paclitaxel plus carboplatin regimen.

#### Numerical solvers

Given that the tumor dynamics in Eq. (1) corresponds to a transient problem while the mechanical equilibrium in Eq. (4) is quasistatic, we adopt a staggered approach to solve the spatiotemporal discretization of the model equations [88, 89]. Since the tumor-driven deformation of the breast tissue during NAC is a slow process and we are solving our model at four time points during every natural day, we solve the quasistatic mechanical equilibrium every four time steps to reduce the computational time required in the simulations of this study [88, 89]. Hence, every four time steps starting at $t=0$, we first solve mechanical equilibrium to update the displacements and then we update the tumor cell density by solving tumor dynamics, while in the remainder time steps we only solve the latter. We solve tumor dynamics by applying the generalized-$\alpha$ method to the spatiotemporal discretization of Eq. (1), which leads to a nonlinear system of equations in each time step [90, 107–109]. We linearize this system using the Newton–Raphson method, and we solve the resulting linear systems by means of the generalized minimal residual (GMRES) method with a diagonal preconditioner [90, 107, 110]. The quasistatic mechanical equilibrium is also solved by directly employing the GMRES algorithm with a diagonal preconditioner on the spatiotemporal discretization of Eq. (4). The integrals involved in the application of the aforementioned solvers to the spatiotemporal discretization of the model are calculated with standard Gaussian quadrature [89, 90, 107]. The numerical methods described in this section and the spatiotemporal discretization of our model were implemented using in-house FORTRAN codes, which were built using the algorithms and recommendations provided in Ref. [107].

### Sensitivity analysis

In this work, we perform a global variance-based sensitivity analysis of our model of breast cancer response to NAC using the approach proposed by Saltelli et al. [50, 111] to calculate the total-effects index $S_{{T},i}$ for each parameter $p_i$ ($i=1,\ldots ,n_{\text {p}}$). This sensitivity metric quantifies the total contribution of variations in each model parameter to the variance of the model outcomes, including both first-order effects and higher-order effects due to interactions with other parameters. As introduced in Sect. 2.3.4, we consider a set of $n_ {p}=15$ model parameters for sensitivity analysis, which are listed in Table 1. To calculate the total-effects indices $S_{{T},i}$, we first generate two independent random samples of the parameter set leveraging Latin Hypercube Sampling as provided by *lhsdesign* in MATLAB. Each of these samples features $n_{s}=1000$ parameter combinations, which are stored in two $n_{s} \times n_{\text {p}}$ matrices $\textbf{A}$ and $\textbf{B}$, respectively. Hence, each row of these matrices represents a parameter combination and each column corresponds to a parameter $p_i$ ($i=1,\ldots ,n_{\text {p}}$). We further generate a total of $n_{\text {p}}$ matrices $\textbf{A}_\textbf{B}^{(i)}$ ($i=1,\ldots ,n_{\text {p}}$) by replacing the *i*-th column from matrix $\textbf{A}$ with the *i*-th column from matrix $\textbf{B}$. Then, we run a total of $n_{s}(n_{\text {p}}+2)$ simulations of our model for each parameter combination in matrices $\textbf{A}$, $\textbf{B}$, and $\textbf{A}_\textbf{B}^{(i)}$, $i=1,\ldots ,n_{\text {p}}$.

In each time step of the model simulations, we use the spatial map of tumor cell density $N(\textbf{x},t)$ to compute two model outcomes of interest: the tumor volume $V_{T}$ and the total tumor cell count $N_{T}$, which are calculated as

16 $$\begin{aligned} V_{T}(t)=\int _\Omega H\left( N(\textbf{x},t)-N_{th}\right) \; {\text {d}}\Omega \end{aligned}$$

and

17 $$\begin{aligned} N_{T}(t)=\int _\Omega N(\textbf{x},t) \; {\text {d}}\Omega . \end{aligned}$$

These two quantities of interest are used ubiquitously in imaging-based computational models of solid tumor growth and treatment response [30, 34, 44, 45, 47, 73–75]. While $V_{T}$ is calculated from the spatial region occupied by the tumor, $N_{T}$ accounts for the heterogeneous spatial distribution of tumor cell density. In Eq. (16), *H* denotes the Heaviside step function and $N_{th}$ is a threshold for the model-calculated tumor cell density to define the tumor segmentation on the simulation results, and ultimately compare it to the corresponding segmentation from the patient’s MRI data [30, 34, 44, 45, 47, 73–75]. Since the natural variations of ADC in healthy breast tissue may overlap with the ADC of areas of low-tumor cell density, $N_{th}$ aims at delineating the simulated tumor region that would be identified as a tumor from the MRI data (i.e., due to significantly low ADC values). Hence, this threshold also enables the calculation of the tumor volume on model simulation results via Eq. (16). Here, we set $N_{th}=\theta /4$, which includes the usual ADC values characterizing breast tumor regions in MRI datasets (e.g., see Fig. 1 and Refs. [44, 45, 47]). By calculating $N_{T}$ over the whole computational domain and $V_{T}$ over a thresholded region, we also assess whether there is a difference in evaluating model simulations using the whole model-calculated tumor cell density map $N(\textbf{x},t)$ or just a restricted region.

For each parameter combination from matrices $\textbf{A}$, $\textbf{B}$, and $\textbf{A}_\textbf{B}^{(i)}$, $i=1,\ldots ,n_{\text {p}}$, we store the value of each model outcome of interest at time *t* in vectors $\textbf{Y}_\textbf{A} (t)$, $\textbf{Y}_\textbf{B} (t)$, and $\textbf{Y}_\textbf{AB}^{(i)} (t)$, respectively, which each have length $n_{s}$. Hence, the values in these vectors can correspond to either tumor cell volume $V_{T}$ or the total tumor cell count $N_{T}$. Finally, the total-effects indices $S_{{T},i} (t)$ for each parameter $p_i$, $i=1,\ldots ,n_{\text {p}}$ on each model outcome of interest at each time step are calculated as

18 $$\begin{aligned} S_{{T},i} (t) = \frac{1}{2 n_{\text {s}} \text {Var}\left( \mathbf {Y_A}(t), \mathbf {Y_B}(t)\right) }\sum _{j=1}^{n_{\text {s}}} \left( \left( \mathbf {Y_A}(t) \right) _j - \left( \mathbf {Y_{AB}}^{(i)}(t) \right) _j \right) ^2 \end{aligned}$$

where $\text {Var}\left( \mathbf {Y_A}(t), \mathbf {Y_B}(t)\right)$ is the variance of all the values of the model outcome of interest at time *t* obtained from all the computer simulations using all the parameter combinations in matrices $\textbf{A}$ and $\textbf{B}$. If $S_{{T},i} = 0$, then the parameter is non-influential and, hence, it can be fixed to any value within the range considered in the sensitivity analysis without affecting the variance of the model output of interest [50]. However, in practice it is common to set a tolerance $\epsilon _{S}$ such that if $S_{{T},i} < \epsilon _{S}$, the parameter $p_i$ is considered non-influential on the model outcomes of interest. Given that the patient-specific imaging data that is available to calibrate our model is scarce (see Sect. 1), we aim to reduce the number of parameters that will require personalized calibration as much as possible, so we set $\epsilon _{s}=0.1$ in the sensitivity analysis of this study. Additionally, 95$\%$ bootstrap confidence intervals for $S_{{T},i}$ at any time *t* can be calculated by resampling the values in $\mathbf {Y_A}(t)$, $\mathbf {Y_B}(t)$, and $\textbf{Y}_\textbf{AB}^{(i)} (t)$ in Eq. (18). In particular, we calculated these 95$\%$ bootstrap confidence intervals at the conclusion of NAC (i.e., at $t=T$) using the MATLAB function *bootci* and 1000 bootstrap samples.

After completing the global sensitivity analysis, we further compare the distribution of the model outcomes of interest (i.e., $V_{T}$ and $N_{T}$) obtained with the original model and the reduced model that results from fixing all non-influential parameters to a value within the parameter space (see Table 1). This comparison is carried out at the time of termination of each NAC regimen (i.e., at $t=T$). Additionally, we conduct a preliminary investigation of the range of heterogeneity in the tumor cell density maps obtained with either model. The ability of a mechanistic model to recapitulate and predict tumor heterogeneity is usually assessed with metrics that compare the spatial tumor morphology obtained with a patient-specific calibrated model and imaging data from the same individual collected at the same timepoint [34, 44–46]. A hypothetical calibration of the original and the reduced model to the same patient-specific longitudinal MRI dataset would produce different values of the dominant parameters (due to the fixed non-influential values in the latter model). However, we do not perform a calibration-forecasting analysis in this work. Furthermore, since the calibrated parameter sets would be different, it would not be fair to compare the tumor cell density maps obtained from the simulations performed with either model for the same choice of dominant parameters. Instead, here we follow a similar analysis as for $V_{T}$ and $N_{T}$, so we define a metric that accounts for heterogeneity in the tumor cell density maps and that can be computed independently with either model. Towards this end, we investigate the heterogeneity of the tumor cell density maps obtained with each model for each parameter combination by assessing the distribution of their mean relative difference (MRD) with the respect to a reference simulation with all parameters fixed. Hence, we can measure the MRD for each model separately with respect to the same reference simulation, and then we compare the distributions of MRD obtained for each model. Agreement between these two distributions would indicate that the reduced model captures the same range of heterogeneity in the tumor cell density maps as the original model. In particular, we choose the reference simulation to represent a no-treatment scenario with the same values of the baseline tumor cell diffusivity and net cell proliferation in both perfusion scenarios (i.e., the reference simulation has unique parameters for all computational scenarios in this study). These parameter values are fixed as $D_0=2\cdot 10^{-4}$ $\text{mm}^{2}/$day and $\rho _0=2.5\cdot 10^{-2}$ 1/day based on our previous work on breast cancer forecasting [44–46]. Then, the MRD is calculated as

19 $$\begin{aligned} {\text {MRD}} (t) = \frac{1}{V_{{T,ref}}(t)}\int _{\Omega _{{\text {ref}}}} \frac{N(\textbf{x},t)-N_{{ref}}(\textbf{x},t)}{N_{{\text {ref}}}(\textbf{x},t)}\; {\text {d}}\Omega , \end{aligned}$$

where $N_{{\text {ref}}}(\textbf{x},t)$ is the tumor cell density map obtained with the reference simulation, $V_{{T,ref}}$ is the volume of the reference tumor calculated using Eq. (16), and $\Omega _{{\text {ref}}}$ is the tumor region in the reference simulation above the threshold $N_{th}$ (i.e., the same as the one used to calculate $V_{{T,ref}}$). As for $V_{T}$ and $N_{T}$, we compare the distribution of MRD obtained for each model at the conclusion of NAC for each treatment regimen and perfusion scenario.

## Results

### Doxorubicin and cyclophosphamide NAC regimen

**Fig. 4:**
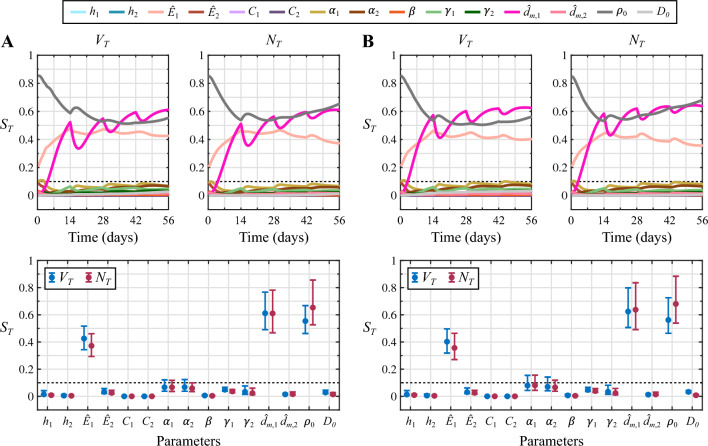
Sensitivity analysis results for the NAC regimen based on doxorubicin and cyclophosphamide. Panel **A** shows the total effects indices ($S_{T}$) for each model parameter and quantity of interest ($V_{T}$ and $N_{T}$) obtained in the well-perfused tumor scenario. Panel **B** provides the same results for the poorly-perfused tumor. The top row in each panel depicts the dynamics of the total effects indices during the course of NAC. The bottom row further shows the corresponding $S_{T}$ values at the conclusion of NAC along with their 95% bootstrapped confidence interval. Parameters with subindex 1 correspond to doxorubicin and parameters with subindex 2 are relative to cyclophosphamide. The dashed line indicates the threshold of the total effects index to consider a parameter as influential ($S_{T}>0.1$) or non-influential ($S_{T}<0.1$). Out of the $n_ {p}=15$ model parameters considered in the sensitivity analysis, only three of them dominate the response of both tumors to the NAC regimen based on doxorubicin and cyclophosphamide: the baseline tumor cell net proliferation ($\rho _0$), the normalized maximal effect of doxorubicin (${\hat{E}}_1$), and the normalized maximal drug concentration of doxorubicin (${\hat{d}}_{m,1}$). Additionally, these observations hold for the two quantities of interest considered in this study ($V_{T}$ and $N_{T}$), whose corresponding $S_{T}$ values for each parameter show similar dynamics and terminal values

Figure 4 presents the results of the global variance-based sensitivity analysis corresponding to the NAC regimen based on doxorubicin and cyclophosphamide in the well-perfused and the poorly-perfused TNBC cases. In both tumor scenarios, only three out of the $n_ {p}=15$ model parameters included in the sensitivity analysis exhibit a total effects index $S_{T}$ on tumor volume ($V_{T}$) and total tumor cell count ($N_{T}$) over the threshold to identify influential parameters ($S_{T}>0.1$): the baseline tumor cell net proliferation ($\rho _0$), the normalized maximal effect of doxorubicin (${\hat{E}}_1$), and the normalized maximal drug concentration of doxorubicin (${\hat{d}}_{m,1}$). This observation holds consistently both during the vast majority of the NAC regimen and at the conclusion of the treatment, when the corresponding 95% confidence intervals of the total effects indices on $V_{T}$ and $N_{T}$ of the three influential parameters are also completely above the $S_{T}$ threshold. As shown in Fig. 4, while $\rho _0$ and ${\hat{E}}_1$ are identified as driving parameters since the onset of the NAC, ${\hat{d}}_{m,1}$ becomes influential during the first cycle. Additionally, each cycle of this NAC regimen alters the rank of the total effects index values on tumor volume ($V_{T}$) and total tumor cell counts ($N_{T}$) of the three influential parameters. According to the global dynamics of the total effects indices on the tumor volume $V_{T}$ over the whole NAC regimen, $\rho _0$ is the most influential parameter at the beginning of treatment, but ${\hat{d}}_{m,1}$ progressively becomes the dominant model parameter towards the conclusion of NAC in both tumor scenarios. The global dynamics of the total effects indices on the total tumor cell count $N_{T}$ show that $\rho _0$ is the most influential parameter over the majority of this NAC regimen and at the conclusion of the treatment in the two tumor cases, only momentarily superseded by ${\hat{d}}_{m,1}$ around the date of delivery of each NAC cycle. Furthermore, following the delivery of the drug cycles at $t=14, 28,$ and 42 days in both tumor scenarios, we observe that the values of the total effects indices $S_{T}$ on $V_{T}$ and $N_{T}$ for parameter ${\hat{d}}_{m,1}$ drop while there is an increase of the corresponding $S_{T}$ values for parameters ${\hat{E}}_1$ and $\rho _0$. However, the total effects indices $S_{T}$ on $V_{T}$ and $N_{T}$ of parameter ${\hat{d}}_{m,1}$ then exhibits an increasing trend during each of these three last cycles, and may ultimately supersede the corresponding $S_{T}$ values for parameters ${\hat{E}}_1$ and $\rho _0$. Among the non-influential parameters in both tumor scenarios, the total effects indices $S_{T}$ on $V_{T}$ and $N_{T}$ corresponding to the coefficients of synergy of potency of both doxorubicin and cyclophosphamide ($\alpha _1$ and $\alpha _2$, respectively) reach values close to $S_{T}=0.1$ according to the results provided in Fig. 4. Indeed, a fraction of their corresponding 95% confidence interval at the conclusion of the NAC regimen is above this $S_{T}$ threshold. Additionally, the total effects indices $S_{T}$ on $V_{T}$ and $N_{T}$ corresponding to the normalized maximal effect of cyclophosphamide (${\hat{E}}_2$) and to the decay rates of doxorubicin and cyclophosphamide ($\gamma _1$ and $\gamma _2$) also take values relatively close to the $S_{T}=0.1$ threshold according to the 95% confidence intervals in both tumor scenarios. Furthermore, this last observation also applies for the total effects index on $V_{T}$ of the baseline tumor cell diffusivity ($D_0$).

**Fig. 5:**
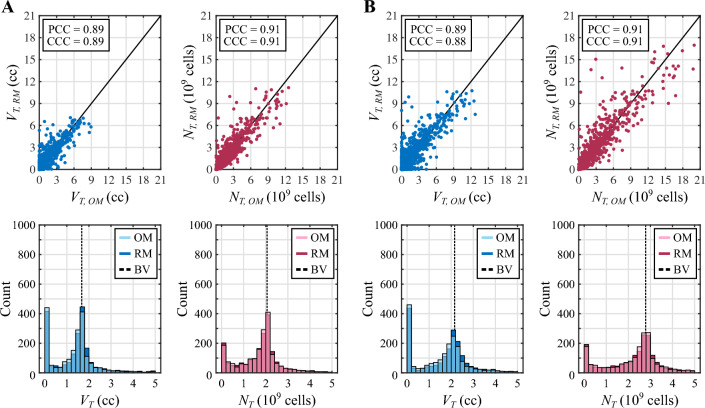
Comparison of the $V_{T}$ and $N_{T}$ distributions from the original and reduced models for the NAC regimen based on doxorubicin and cyclophosphamide. Panel **A** compares the distribution of the tumor volume ($V_{T}$, blue) and the total tumor cell count ($N_{T}$, magenta) at the termination of NAC obtained using the original model (OM) and the reduced model (RM) in the well-perfused tumor. Panel **B** provides the corresponding results for the poorly-perfused tumor. In each panel, the unity plots in the top row show the correlation between the $V_{T}$ and $N_{T}$ values obtained with either model ($n=2000$ for each quantity of interest and tumor scenario). This correlation is further quantified in terms of the Pearson and the concordance correlation coefficients (PCC and CCC, respectively). Additionally, the histograms in the bottom row of each panel compare the distribution of $V_{T}$ and $N_{T}$ values obtained with the original and reduced models in either tumor scenario. A dashed vertical line indicates the baseline value (BV) of $V_{T}$ and $N_{T}$ at $t=0$ for each tumor, as a reference to assess NAC efficacy. To facilitate the visualization of the main region of these distributions only the values of $V_{T}$ in [0, 5] cc and $N_{T}$ in $[0,5]\cdot 10^9$ cells are represented, which account for more than 90% of the values obtained for each quantity of interest in either tumor (i.e., these plots do not depict higher values corresponding to outliers). The plots depicted in this figure show good qualitative and quantitative agreement between the original and reduced models for the same choice of influential parameter values. Importantly, these results further demonstrate that the solution space of both models contains a similar distribution of NAC outcomes, ranging from pCR to progressive disease

**Fig. 6:**
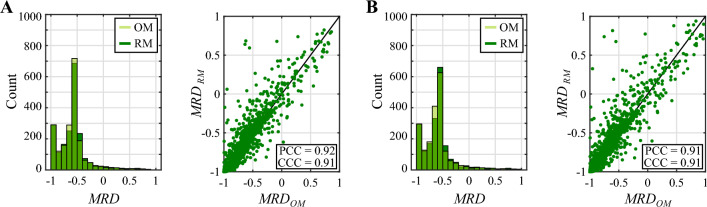
Comparison of the MRD distributions from the original and reduced models for the NAC regimen based on doxorubicin and cyclophosphamide. This figure compares the distribution of the mean relative difference (MRD) of the tumor cell density maps obtained using the original model (OM) and the reduced model (RM) with respect to the reference solution at the termination of NAC. While panel **A** presents the results for the well-perfused tumor, panel **B** provides the corresponding results for the poorly-perfused tumor. In each panel, the histograms compare the distribution of MRD values obtained with the original and reduced models in either tumor scenario. Additionally, the unity plots show the correlation between the MRD values obtained with either model ($n=2000$ for each tumor scenario). This correlation is further quantified in terms of the Pearson and the concordance correlation coefficients (PCC and CCC, respectively). The plots depicted in this figure show good qualitative and quantitative agreement between the original and reduced models for the same choice of influential parameter values. Importantly, these results further suggest that both models are able to represent the same range of tumor heterogeneity in the tumor cell density maps calculated at the conclusion of NAC

**Fig. 7:**
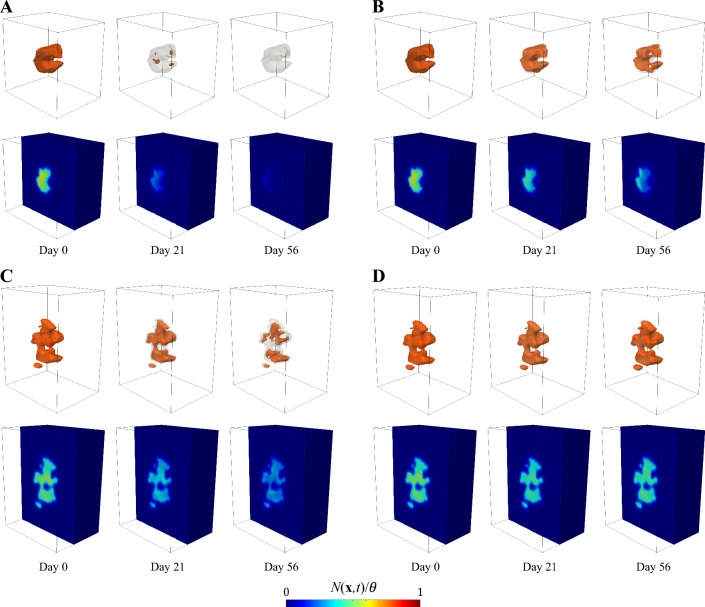
Examples of breast cancer response to the NAC regimen based on doxorubicin and cyclophosphamide. Panels **A** and **B** illustrate two simulations of the response of the well-perfused tumor at the beginning (day 0), the middle of the second cycle (day 21), and the conclusion of NAC (day 56). Likewise, panels **C** and **D** provide two examples of the response of the poorly-perfused tumor at the same three time points. Each simulation corresponds to a choice of the model parameters within the bounds set in Table 1 (see Supplementary Table S5). In each panel, the top row represents the outline of the tissue box including the evolving geometry of the tumor volume (orange) at the three time points, which is delineated over the model simulation results as $N(\textbf{x},t)>N_{th}$ with $N_{th}=\theta /4$. These images further show the baseline tumor geometry at $t=0$ (gray volume) as a reference to assess the change in the tumor volume over the course of NAC. The bottom row in each panel shows corresponding sagittal sections of the tumor cell density map $N(\textbf{x},t)$. The response scenario in panel **A** represents a pCR case, panels **B** and **C** provide mild responses to this NAC regimen, and panel **D** shows a non-responder scenario. Notice that the status of the tumor by the middle of the second cycle is already informative of the expected therapeutic outcome at the conclusion of NAC. We remark that this figure only provides two examples of response to the NAC regimen based on doxorubicin and cyclophosphamide for each of the two tumors considered in this study. However, as shown in Fig. 5, there are multiple model parameter combinations yielding responses to this NAC regimen that range from pCR to progressive disease for both tumors

The sensitivity analysis results for the NAC regimen based on doxorubicin and cyclophosphamide justify the definition of a reduced model where only $\rho _0$, ${\hat{E}}_1$, and ${\hat{d}}_{m,1}$ need to be identified for each individual patient, while the rest of the model parameters can be fixed to any value within the ranges in Table 1. Figure 5 compares the distribution of tumor volumes and total tumor cell counts ($V_{T}$ and $N_{T}$, respectively) obtained with the original and the reduced model at the conclusion of NAC in both tumor scenarios using the parameter combinations in matrices $\textbf{A}$ and $\textbf{B}$ that were leveraged during the sensitivity analysis ($n=2000$ parameter combinations; see Sect. 2.5). Hence, for the reduced model, only the values corresponding to $\rho _0$, ${\hat{E}}_1$, and ${\hat{d}}_{m,1}$ are varied, while the rest of the parameters are fixed to the values reported in Supplementary Table S3 for all parameter combinations. Figure 5 shows that the values of tumor volume ($V_{T}$) and total tumor cell count ($N_{T}$) obtained with either model at the end of the NAC regimen exhibit a high correlation in both tumor scenarios. In particular, the Pearson and concordance correlation coefficients (PCC and CCC, respectively) for the $V_{T}$ values obtained with the original and the reduced model are both 0.89 in the well-perfused tumor, and 0.89 and 0.88 in the poorly-perfused tumor, respectively. Similarly, the PCC and CCC of the $N_{T}$ values obtained with either model version are 0.91 in both tumor scenarios. Figure 5 further provides a detail of the main part of the distribution (i.e., >90% of values in the sample) of $V_{T}$ and $N_{T}$ values obtained with the original and the reduced model in the well-perfused and the poorly-perfused tumor. These snapshots show that the distributions of tumor volumes ($V_{T}$) and total tumor cell counts ($N_{T}$) obtained with either model are very similar. Importantly, these distributions also show that both models can successfully recapitulate the diverse spectrum of TNBC responses to NAC based on doxorubicin and cyclophosphamide, ranging from a complete tumor eradication (i.e., pCR) to treatment failure exhibiting a larger tumor with respect to the corresponding baseline measurement (i.e., at $t=0$). Additionally, Fig. 6 compares the distribution of the mean relative difference (MRD) of the tumor cell density maps obtained with either model with respect to the reference solution at the end of NAC. We observe that the values obtained for this metric with the original and the reduced model exhibit a very similar distribution and high correlation. In particular, we obtained a PCC of 0.92 and 0.91 in the well-perfused and the poorly-perfused scenario, respectively. The CCC is 0.91 in both cases. Thus, these results for MRD indicate that both models are able to reproduce the same range of spatial heterogeneity in the tumor cell density maps obtained at the conclusion of NAC. To further illustrate this diversity of TNBC responses to a doxorubicin and cyclophosphamide NAC regimen, Fig. 7 shows four examples of the 3D dynamics of the tumors during this neoadjuvant regimen including a pCR, two mild responses, and treatment failure. In particular, this figure shows both the change in the 3D volume of the tumor and the local map of tumor cell density (i.e., $N(\textbf{x},t)$) halfway through the second NAC cycle ($t=21$ days) and at the end of treatment ($t=56$ days) with respect to baseline ($t=0$). We notice that, in the cases where there is a certain level of response (i.e, Fig. 7A–C), this phenomenon is more noticeable as a reduction of the values of the tumor cell density map than as a reduction of the tumor volumetric region. This observation supports the use of MRI measurements and computational predictions of tumor cell density maps $N(\textbf{x},t)$ to characterize TNBC response to NAC rather than traditional tumor length or volume measurements.

### Paclitaxel and carboplatin NAC regimen

**Fig. 8:**
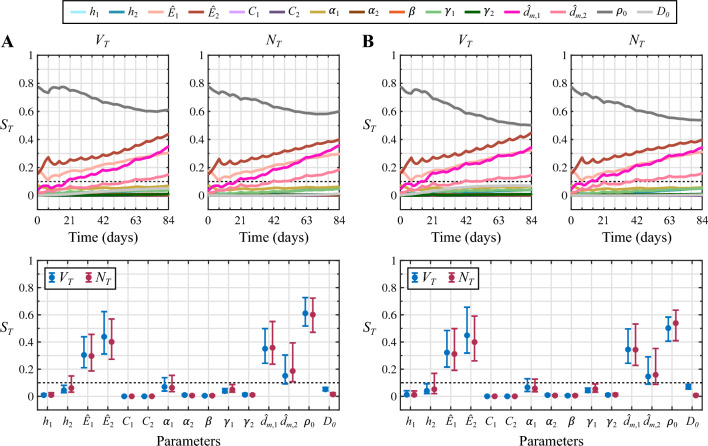
Sensitivity analysis results for the NAC regimen based on paclitaxel and carboplatin. Panel **A** shows the total effects indices ($S_{T}$) for each model parameter and quantity of interest ($V_{T}$ and $N_{T}$) obtained in the well-perfused tumor scenario. Panel **B** provides the same results for the poorly-perfused tumor. The top row in each panel depicts the dynamics of the total effects indices during the course of NAC. The bottom row further shows the corresponding $S_{T}$ values at the conclusion of NAC along with their 95% bootstrapped confidence interval. Parameters with subindex 1 correspond to paclitaxel and parameters with subindex 2 are relative to carboplatin. The dashed line indicates the threshold of the total effects index to consider a parameter as influential ($S_{T}>0.1$) or non-influential ($S_{T}<0.1$). Out of the $n_ {p}=15$ model parameters considered in the sensitivity analysis, we observe that only five of them dominate the response of both tumors to the NAC regimen based on paclitaxel and carboplatin: the baseline tumor cell net proliferation ($\rho _0$), the normalized maximal effects of both paclitaxel and carboplatin (${\hat{E}}_1$ and ${\hat{E}}_2$), and the normalized maximal drug concentration of both paclitaxel and carboplatin (${\hat{d}}_{m,1}$ and ${\hat{d}}_{m,2}$). Additionally, these observations hold for the two quantities of interest considered in this study ($V_{T}$ and $N_{T}$), whose corresponding $S_{T}$ values for each parameter show similar dynamics and terminal values

Figure 8 provides the results of the global variance-based sensitivity analysis corresponding to the NAC regimen based on paclitaxel and carboplatin in the well-perfused and the poorly-perfused TNBC scenarios. In both cases, only five out the $n_ {p}=15$ model parameters considered in the sensitivity analysis reach values of the total effects index ($S_{T}$) on tumor volume ($V_{T}$) and total tumor cell count ($N_{T}$) over the threshold to identify influential parameters ($S_{T}>0.1$): the baseline tumor cell net proliferation ($\rho _0$), the normalized maximal effects of both paclitaxel and carboplatin (${\hat{E}}_1$ and ${\hat{E}}_2$), and the normalized maximal drug concentration of both paclitaxel and carboplatin (${\hat{d}}_{m,1}$ and ${\hat{d}}_{m,2}$). However, not all these parameters are classified as influential during the whole course of the paclitaxel and carboplatin NAC regimen. While $\rho _0$, ${\hat{E}}_1$, and ${\hat{E}}_2$ exhibit $S_{T}>0.1$ on both $V_{T}$ and $N_{T}$ from the beginning of the NAC regimen, the corresponding total effects indices for ${\hat{d}}_{m,1}$ only become influential by the end of the first NAC cycle and for ${\hat{d}}_{m,2}$ by the end of the second or third cycle (see Fig. 8). At the conclusion of the NAC regimen, the five parameters are classified as influential and their corresponding 95% confidence intervals are above the $S_{T}=0.1$ threshold, except for a minimal fraction for ${\hat{d}}_{m,1}$ and ${\hat{d}}_{m,2}$. Regarding the rank of the driving parameters, we observe that $\rho _0$ and ${\hat{E}}_2$ are consistently identified as the first and second most influential parameters according to the total effects indices on tumor volume ($V_{T}$) and total tumor cell count ($N_{T}$) in both tumor scenarios, while ${\hat{d}}_{m,2}$ always shows the lowest $S_{T}$ values among the five influential parameters detected for this NAC regimen. Additionally, while ${\hat{E}}_1$ is more influential than ${\hat{d}}_{m,1}$ at the beginning of treatment, their rank is reversed towards the conclusion of the NAC regimen. This last observation also holds both for the total effects indices on tumor volume and total tumor cell count as well as for both tumor scenarios. Figure 8 further shows that the weekly delivery of paclitaxel introduces local peaks or minima in the values of the total effects indices on tumor volume ($V_{T}$) and total tumor cell count ($N_{T}$), but that the overall dynamics during the whole course of this NAC regimen further account for the action of carboplatin (see Fig. 3). Among the non-influential parameters in both tumor scenarios, the total effects indices $S_{T}$ of $V_{T}$ and $N_{T}$ of the Hill coefficient of carboplatin ($h_2$), the decay rate of paclitaxel ($\gamma _1$), and the coefficient of synergy of potency of paclitaxel ($\alpha _1$) are close to the $S_{T}$ threshold by the end of treatment, and a fraction of their 95% confidence interval may even be above the $S_{T}=0.1$ line (see Fig. 4). Additionally, the total effects index on $V_{T}$ of the baseline tumor cell diffusivity ($D_0$) also reaches values near the $S_{T}=0.1$ threshold in both tumor scenarios.

**Fig. 9:**
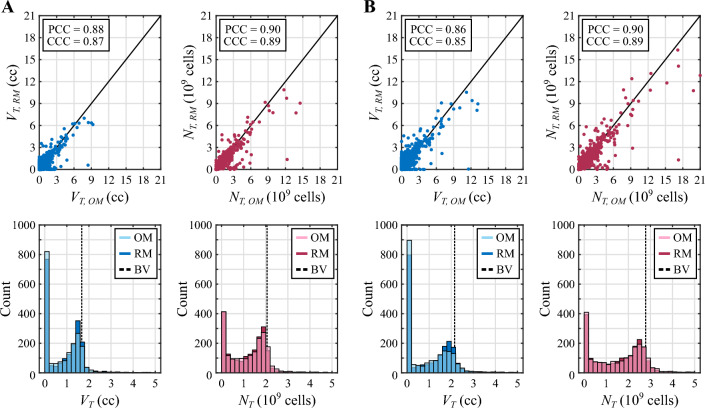
Comparison of the $V_{T}$ and $N_{T}$ distributions from the original and reduced models for the NAC regimen based on paclitaxel and carboplatin. Panel **A** compares the distribution of the tumor volume ($V_{T}$, blue) and the total tumor cell count ($N_{T}$, magenta) at the termination of NAC obtained using the original model (OM) and the reduced model (RM) in the well-perfused tumor. Panel **B** provides the corresponding results for the poorly-perfused tumor. In each panel, the unity plots in the top row show the correlation between the $V_{T}$ and $N_{T}$ values obtained with either model ($n=2000$ for each quantity of interest and tumor scenario). This correlation is further quantified in terms of the Pearson and the concordance correlation coefficients (PCC and CCC, respectively). Additionally, the histograms in the bottom row of each panel compare the distribution of $V_{T}$ and $N_{T}$ values obtained with the original and reduced models in either tumor scenario. A dashed vertical line indicates the baseline value (BV) of $V_{T}$ and $N_{T}$ at $t=0$ for each tumor, as a reference to assess NAC efficacy. To facilitate the visualization of the main region of these distributions only the values of $V_{T}$ in [0, 5] cc and $N_{T}$ in $[0,5]\cdot 10^9$ cells are represented, which account for more than 90% of the values obtained for each quantity of interest in either tumor (i.e., these plots do not depict higher values corresponding to outliers). The plots depicted in this figure show good qualitative and quantitative agreement between the original and reduced models for the same choice of influential parameter values. Importantly, these results further demonstrate that the solution space of both models contains a similar distribution of NAC outcomes, ranging from pCR to progressive disease

**Fig. 10:**
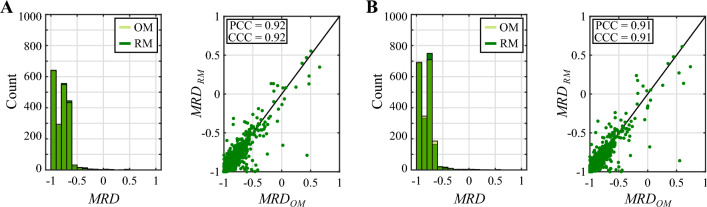
Comparison of MRD distributions from the original and reduced models for the NAC regimen based on paclitaxel and carboplatin. This figure compares the distribution of the mean relative difference (MRD) of the tumor cell density maps obtained using the original model (OM) and the reduced model (RM) with respect to the reference solution at the termination of NAC. While panel **A** presents the results for the well-perfused tumor, panel **B** provides the corresponding results for the poorly-perfused tumor. In each panel, the histograms compare the distribution of MRD values obtained with the original and reduced models in either tumor scenario. Additionally, the unity plots show the correlation between the MRD values obtained with either model ($n=2000$ for each tumor scenario). This correlation is further quantified in terms of the Pearson and the concordance correlation coefficients (PCC and CCC, respectively). The plots depicted in this figure show good qualitative and quantitative agreement between the original and reduced models for the same choice of influential parameter values. Importantly, these results further suggest that both models are able to represent the same range of tumor heterogeneity in the tumor cell density maps calculated at the conclusion of NAC

**Fig. 11:**
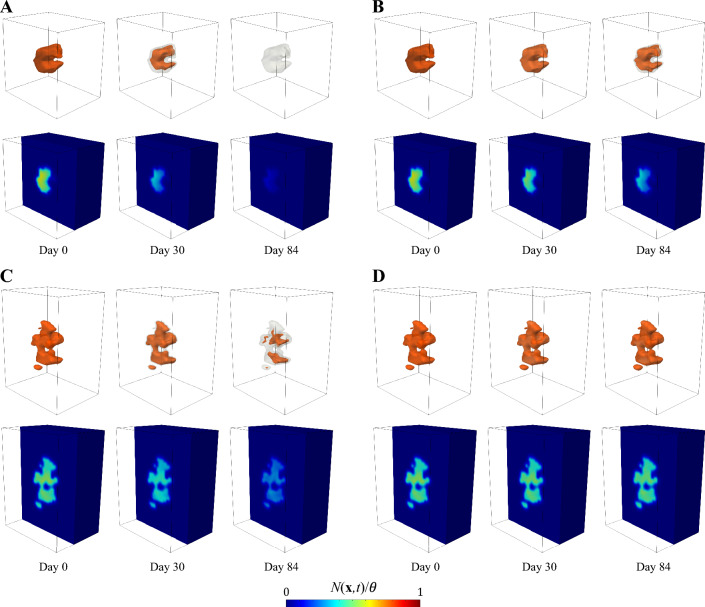
Examples of breast cancer response to the NAC regimen based on paclitaxel and carboplatin. Panels **A** and **B** illustrate two simulations of the response of the well-perfused tumor at the beginning (day 0), the middle of the second cycle (day 30), and the conclusion of NAC (day 84). Likewise, panels **C** and **D** provide two examples of the response of the poorly-perfused tumor at the same three time points. Each simulation corresponds to a choice of the model parameters within the bounds set in Table 1 (see Supplementary Table S6). In each panel, the top row represents the outline of the tissue box including the evolving geometry of the tumor volume (orange) at the three time points, which is delineated over the model simulation results as $N(\textbf{x},t)>N_{th}$ with $N_{th}=\theta /4$. These images further show the baseline tumor geometry at $t=0$ (gray volume) as a reference to assess the change in the tumor volume over the course of NAC. The bottom row in each panel shows corresponding sagittal sections of the tumor cell density map $N(\textbf{x},t)$. The response scenario in panel **A** represents a pCR case, panels **B** and **C** provide mild responses to this NAC regimen, and panel **D** shows a non-responder scenario. Notice that the status of the tumor by the middle of the second cycle is already informative of the expected therapeutic outcome at the conclusion of NAC. We remark that this figure only provides two examples of response to the NAC regimen based on paclitaxel and carboplatin for each of the two tumors considered in this study. However, as shown in Fig. 9, there are multiple model parameter combinations yielding responses to this NAC regimen that range from pCR to progressive disease for both tumors

Based on the results of the sensitivity analysis for the paclitaxel and carboplatin NAC regimen, we can define a reduced model where only $\rho _0$, ${\hat{E}}_1$, ${\hat{E}}_2$, ${\hat{d}}_{m,1}$, and ${\hat{d}}_{m,2}$ require patient-specific identification, while the rest of the model parameters can be fixed to any value within the ranges defined in Table 1. Figure 9 provides a comparison between the distribution of tumor volumes ($V_{T}$) and total tumor cell counts ($N_{T}$) obtained with the original and the reduced model at the termination of this NAC regimen in the well-perfused and the poorly-perfused tumor scenario. Again, we leveraged the parameter combinations in matrices $\textbf{A}$ and $\textbf{B}$ of the sensitivity analysis ($n=2000$; see Sect. 2.5). Hence, for the reduced model we only vary the values of $\rho _0$, ${\hat{E}}_1$, ${\hat{E}}_2$, ${\hat{d}}_{m,1}$, and ${\hat{d}}_{m,2}$ while the rest of the parameters are fixed to the values provided in Supplementary Table S4. According to the unity plots presented in Fig. 9, the values of tumor volume ($V_{T}$) and total tumor cell count ($N_{T}$) obtained with either model at the conclusion of NAC are highly correlated in both tumor scenarios. In particular, the PCC and CCC for the $V_{T}$ values obtained with the original and the reduced model in the well-perfused tumor are 0.88 and 0.87, respectively, while in the poorly-perfused tumor the corresponding PCC and CCC values are 0.86 and 0.85, respectively. Likewise, the PCC and CCC of the $N_{T}$ values obtained with either model version are 0.90 and 0.89, respectively, in both the well-perfused and the poorly-perfused scenarios. Figure 9 also shows a detail of the main part of the distribution (i.e., >90% of values in the sample) of the $V_{T}$ and $N_{T}$ values obtained with the original and the reduced model in each tumor scenario. We observe that these distributions exhibit good qualitative agreement, demonstrating that our modeling framework can successfully reproduce the different responses of TNBC to an NAC regimen based on paclitaxel and carboplatin, ranging from the complete elimination of the tumor to progressive disease by the end of treatment. Additionally, Fig. 10 provides a comparison of the distribution of the mean relative difference (MRD) of the tumor cell density maps calculated with each model with respect to the reference solution at NAC conclusion. As for the other NAC regimen, the values for this metric obtained with the original and the reduced model show a very similar distribution and high correlation. In particular, the PCC and CCC were both 0.92 in the well-perfused scenario and both 0.91 in the poorly-perfused scenario. Thus, based on these results for MRD, both models can equally reproduce the same range of spatial heterogeneity in the tumor cell density maps obtained at the conclusion of NAC. Furthermore, Fig. 11 provides illustrative examples of the 3D dynamics of TNBC response to this NAC regimen, including a pCR, two mild responses, and a treatment failure. In particular, this figure depicts both the 3D tumor region and the local map of tumor cell density (i.e., $N(\textbf{x},t)$) at baseline ($t=0$ days), halfway during the second NAC cycle ($t=30$ days), and at the conclusion of treatment ($t=84$ days). As for doxorubicin and cyclophosphamide, we observe that a positive TNBC response to the paclitaxel and carboplatin NAC regimen (e.g., see Fig. 11A–C) is more noticeable as a reduction of the local values of $N(\textbf{x},t)$ rather than a decrease in the tumor region. Hence, this observation also supports the use of MRI measurements and computational forecasts of tumor cell density maps to assess TNBC response to NAC over conventional metrics relying on tumor length or volume.

## Discussion

NAC has been widely regarded as an opportunity to optimize the management of TNBC for individual patients by adjusting their treatment based on the early tumor response to the initially prescribed drug regimen [1, 10–12, 45, 47]. Towards this end, the integration of longitudinal MRI measurements from an individual patient within mechanistic models of breast cancer growth has shown promise in predicting therapeutic outcome at the conclusion of NAC via personalized computer simulations [44–47, 73, 74]. In this context, we have presented a mechanistic model that describes the spatiotemporal dynamics of breast cancer response to NAC. Our model characterizes tumor growth in terms of tumor cell density using an established formulation consisting of a combination of net proliferation, via a logistic term, and tumor cell mobility, via a mechanically-constrained diffusion operator [30, 44, 45, 47, 73, 74]. Additionally, we propose to model the tumor response to NAC by adjusting the baseline tumor cell net proliferation rate using a term that integrates the NAC drug pharmacokinetics and pharmacodynamics, including potential drug–drug synergies according to the MuSyC framework [57]. We also performed a global variance-based sensitivity analysis to identify the dominant parameters that represent the model mechanisms driving breast cancer response to two common NAC regimens: doxorubicin plus cyclophosphamide, and paclitaxel plus carboplatin [3, 12, 45, 66]. Towards this end, we constrained the parameter space of our model by integrating in vitro measurements of the response of TNBC cell lines to the two aforementioned NAC drug combinations and in silico estimates of TNBC dynamics during these NAC regimens from personalized forecasts informed by in vivo quantitative MRI data. Since the NAC drugs considered in this study are delivered intravenously, we further performed the sensitivity analysis in two different scenarios corresponding to a well-perfused and a poorly perfused tumor. These two tumor scenarios were constructed by leveraging quantitative MRI data from two TNBC patients, respectively.

The global sensitivity analysis resulted in values of the total effects indices ($S_{T}$) on tumor volume ($V_{T}$) and total tumor cell count ($N_{T}$) showing that only a minority of the considered model parameters ($n_ {p}=15$) dominate the response of the tumor cases to the two NAC regimens investigated in this study ($S_{T}>0.1$): three in the case of doxorubicin plus cyclophosphamide, and five in the case of paclitaxel plus carboplatin (see Figs. 4 and 8). The baseline tumor cell proliferation rate ($\rho _0$) was always identified as an influential parameter, and in the paclitaxel and carboplatin NAC regimen it represents the primary model mechanism driving the tumor response over the whole course of the treatment. Additionally, our sensitivity analysis also identified the normalized maximal effects (${\hat{E}}$) and the normalized maximal drug concentration (${\hat{d}}_{m}$) of doxorubicin, paclitaxel, and carboplatin as dominant parameters. No model parameter related to cyclophosphamide was identified as relevant in this study (see Fig. 4). This might be due to the relatively fast wash-out rate of this drug compared to doxorubicin [99, 102–104]. Hence, our sensitivity analysis results suggest that it suffices to fix the parameters characterizing the pharmacodynamics and pharmacokinetics of cyclophosphamide within the ranges of Table 1. Notice that this result does not conclude that cyclophosphamide can be eliminated from the standard NAC regimen, but rather that the formulation of cyclophosphamide effects in the reduced model can be simplified. Indeed, the calibration of the reduced model to longitudinal data from TNBCs treated with doxorubicin only *versus* doxorubicin and cyclophosphamide can lead to different distributions of the calibrated values of the parameters describing doxorubicin effects within the limits defined in Table 1. These differences would emanate from the addition of cyclophosphamide, which may condition the doxorubicin parameters to take values on a different subregion of the parameter space than in the case of NAC based only on doxorubicin. Hence, future studies can leverage our model to quantify the gain in therapeutic outcomes and investigate the changes in the parameterization of the reduced model related to the addition of cyclophosphamide to a baseline NAC based on doxorubicin. Taken together the dominant model parameters, our sensitivity analysis concludes that drug-induced changes of the baseline tumor cell net proliferation constitute the dominant model mechanism characterizing TNBC response to the NAC regimens investigated herein. Our results further indicate that these changes are ultimately driven by the maximum drug concentration reaching the tumor and the maximum drug-induced cytotoxic effect on the baseline tumor cell net proliferation. Importantly, the global sensitivity analysis identifies the same dominant parameters for each NAC regimen in both perfusion scenarios. This result indicates that, although absolute drug effects to tumor dynamics may differ in either perfusion scenario (see $V_{T}$ and $N_{T}$ distributions in Figs. 5 and 9), they are always the dominant model mechanism contributing to the total variance of model outcomes (e.g., tumor volumes and total tumor cell counts). Additionally, our results support the use of longitudinal MRI measurements of tumor cellurarity obtained via DW-MRI to assess tumor response to NAC, given that drug-induced changes of tumor cell net proliferation result in local variations of the tumor cell density according to our model (see Sect. 2.3.1) [30, 44, 45, 47, 73, 74]. Indeed, the 3D simulations provided in Figs. 7 and 11 show that the NAC-induced reduction in tumor cell density renders more insight into the therapeutic response of TNBC than the change of the tumor volume region.

To assess the ability of the dominant model parameters in driving TNBC response to NAC, we further compared the distribution of the tumor volume ($V_{T}$) and the total tumor cell count ($N_{T}$) at NAC conclusion obtained with our original model for the parameter combinations used in the sensitivity analysis ($n=2000$) to the corresponding distributions of these quantities of interest obtained with a reduced version of the model, in which only the dominant parameters are varied while the non-influential parameters are fixed to values within the ranges in Table 1 (see Supplementary Tables S3 and S4). This analysis showed a high correlation between the $V_{T}$ values obtained with either model, which reached a PCC>0.86 and CCC$>0.85$, as well as between the corresponding $N_{T}$ values, for which PCC>0.90 and CCC$>0.89$, across both tumor scenarios and NAC regimens (see Figs. 5 and 9). Additionally, Figs. 5, 7, 9, and 11 show that both versions of the model produce distributions of $V_{T}$ and $N_{T}$ values that are qualitatively similar, ranging from tumor elimination (i.e., pCR) to progressive disease, where the tumor burden is larger than at the onset of NAC. These results demonstrate that the parameter space constructed in this study by integrating in vivo, in vitro, and in silico data of TNBC response to NAC contains parameter combinations that enable both the original and reduced models to span tumor responses across the diverse spectrum observed in clinical practice [7, 9, 17, 18, 45]. Furthermore, we investigated the range of the heterogeneity in the tumor cell density maps that each model can produce at the end of NAC using the parameter combinations of the global sensitivity analysis regimens. To this end, we assessed their mean relative difference (MRD) with respect to the tumor cell density map of a reference simulation with no treatment. This analysis showed high similarity and correlation between the MRD distributions of both models in both perfusion scenarios and NAC regimens, achieving PCC and CCC values over 0.90. These results suggest that the reduced model can represent the same range of heterogeneity in the tumor cell density maps as the original model. Thus, the quantitative and qualitative agreement between the original and the reduced model support the use of the reduced model as a surrogate to characterize the dynamics of TNBC response to NAC in individual patients. Importantly, the reduced parameter set requiring patient-specific identification ($n_{\text {p}}=3$ or 5, depending on the NAC regimen, *versus* $n_ {p}=15$ in the original model) constitutes a practical advantage to deploy our predictive modeling framework in the neoadjuvant setting due to the scarcity of longitudinal MRI data on therapeutic response for each individual patient during the course of NAC, which can only reliably inform a limited number of model parameters.

From a modeling perspective, the choice of the values for the non-influential parameters can introduce an important simplification in the model formulation. For both drugs in the two NAC regimens in this study, the Hill coefficients ($h_1$ and $h_2$) and the coefficients of drug synergy of potency ($\alpha _1$ and $\alpha _2$) are classified as non-influential in the sensitivity analysis ($S_{T}<0.1$). If we select the values of these four parameters within the ranges in Table 1 such that $\alpha _2^{h_1}=\alpha _1^{h_2}$, then $g_0\left( {\hat{d}}_1,{\hat{d}}_2\right) =g_1\left( {\hat{d}}_1,{\hat{d}}_2\right) =g_2\left( {\hat{d}}_1,{\hat{d}}_2\right) =g_3\left( {\hat{d}}_1,{\hat{d}}_2\right) /\alpha _1^{h_2}$ in Eqs. (9)–(12) and, hence, Eq. (8) can be simplified to

20 $$\begin{aligned} \rho = \rho _0 \frac{ 1 + {\hat{d}}_1^{h_1}{\hat{E}}_1 + {\hat{d}}_2^{h_2}{\hat{E}}_2 + \alpha _1^{h_2}{\hat{d}}_1^{h_1}{\hat{d}}_2^{h_2}{\hat{E}}_3}{ 1 + {\hat{d}}_1^{h_1} + {\hat{d}}_2^{h_2} + \alpha _1^{h_2}{\hat{d}}_1^{h_1}{\hat{d}}_2^{h_2} }, \end{aligned}$$

where ${\hat{E}}_3$ is provided by Eq. (13). The relationship $\alpha _2^{h_1}=\alpha _1^{h_2}$ implies that the drugs are in detailed balance [57, 60], which ultimately assumes that the cytotoxic effect of the drugs in each NAC regimen is instantaneous. This particular case for the MuSyC equation was also considered in the study where this modeling framework was presented [57]. Importantly, the sensitivity analysis results from our work support the adoption of this assumption for TNBC forecasting during NAC regimens based on either doxorubicin and cyclophosphamide or paclitaxel and carboplatin. Hence, the simpler formulation of Eq. (20) facilitates the mechanistic analysis and code implementation of our mechanistic model of TNBC response to NAC.

While the sensitivity analysis and ensuing modeling study presented in this work shows promise for the use of our modeling framework to render personalized tumor forecasts of TNBC response to NAC, it also features several limitations. First of all, the construction of the parameter space of our mechanistic model relies on four TNBC cell lines and two patient-specific MRI datasets. These data sources enabled the construction of a broad parameter space, in which each parameter combination represents a distinct manifestation of TNBC. However, these data sources cannot capture the overall landscape of tumor phenotypes, perfusion, size, shape, and cellular density that can be observed in TNBC. At the same time, performing a global variance-based sensitivity analysis over a large cohort exhibiting the wide biological diversity of TNBC would be computationally prohibitive. Furthermore, the mechanistic model proposed in this work is built on a previous model that has been shown to successfully recapitulate and predict breast cancer response to NAC in two different cohorts [45, 46]. Thus, we posit that the reduced model obtained in the global sensitivity analysis performed herein constitutes a promising candidate to describe TNBC response to NAC, but its predictive power needs to be evaluated in a fitting-forecasting study over a sufficiently large TNBC patient cohort (e.g., following the approach in [45, 46]). Importantly, this strategy consisting of analyzing new models in a reduced number of scenarios within an initial computational study and then evaluating their predictive power in a clinical-computational study over a patient cohort has proven successful in our previous work [34, 44–46, 48, 67, 73–75, 112]. Moreover, in this work we focused on TNBC and two NAC regimens, but our modeling framework can also be extended to other breast cancer subtypes and neoadjuvant drugs [2, 3, 12, 14]. To this end, our multiscale framework would need representative tumor scenarios using in vivo MRI data characterizing the tumor subtypes. It would also be necessary to define an appropriate parameter space by obtaining in vitro measurements of tumor cell response to the neoadjuvant drugs of interest for each breast cancer subtype. Additionally, the parameter space could also be refined by integrating in silico estimates of model parameters from tumor forecasts for patients exhibiting each subtype and receiving the neoadjuvant drugs under investigation. Furthermore, given that the combination of NAC with the immunotherapeutic drug pembrolizumab has been shown to improve treatment outcomes in TNBC patients [113], our model could be extended to account for this form of neoadjuvant therapy. While a simple approach could consist of including the ultimate tumor cell death rate induced by pembrolizumab in the tumor equation, a more precise modeling strategy would further require accounting for the spatiotemporal dynamics of T cells and how they kill tumor cells [114–116]. Importantly, these futures studies considering alternative neoadjuvant regimens and other breast cancer subtypes should also leverage datasets accounting for patient diversity (e.g., race, ethnicity, age), which has a limited representation in this work since the two MRI datasets and four cell lines come primarily from white women.

A central assumption of our multiscale framework is that the parameter ranges for the synergy coefficients ($\alpha _1,\alpha _2,\beta$), the Hill coefficients ($h_1,h_2$), and the half-maximal effective drug concentrations ($C_1,C_2$) are directly adopted from the analysis of the in vitro experiments on four TNBC cells. This assumption follows from the impossibility to measure these parameters in a practical way during the standard management of TNBC patients. Furthermore, we computationally estimated the ranges for the normalized maximal drug effects (${\hat{E}}_1,{\hat{E}}_2$) for the in vivo organ-scale scenarios considered in this study. The adopted ranges enabled the model to capture NAC outcomes from tumor eradication (pCR) to progressive disease, which would not have been possible if we directly adopted the corresponding values for this parameter from the TNBC cell line measurements. Thus, future studies over diverse patient cohorts could provide insight in the ability of the ranges of the aforementioned parameters to capture TNBC response to NAC, especially for the dominant parameters included in the reduced model for each regimen. Additionally, preclinical studies in three-dimensional in vitro configurations (e.g., tumor spheroids) and in vivo animal models (e.g., patient-derived xenografts) are ideal scenarios enabling the collection of longitudinal data to refine the ranges of the parameters in our mechanistic model governing drug pharmacodynamics.

Another important assumption is that the tissue-box computational domains with averaged mechanical properties suffice to simulate realistic TNBC dynamics during NAC. This computational strategy was chosen to ensure that the large number of simulations of the global sensitivity analysis were computationally tractable (see Sects. 2.1 and 2.5). Nevertheless, the tissue-box simplification comes with two potential limitations to generalize the results of this work to patient-specific breast geometries with non-homogeneous mechanical property maps: (i) boundary effects, which would affect the dynamics of TNBC cases growing up to the vicinity of the tissue box boundary; and (ii) oversimplification of the mechanical inhibition of tumor cell mobility due to the use of homogeneous mechanical properties. Figures 5 and 9 show that the majority of simulations using the parameter samples of the global sensitivity analysis resulted in tumors with moderately larger, similar, or lower volume at NAC termination than the initial tumor in each perfusion scenario. Notice that the histogram counts drop fast right after the dashed line indicating the baseline value of the tumor volume or the total tumor cell counts. This observation suggests that boundary effects have little effect on the results of our global sensitivity analysis, given that the tissue box was constructed to include a ring of healthy tissue around the initial tumor to avoid them (see Figs. 1, 7, and 11). Additionally, the mechanical inhibition of tumor dynamics is controlled by the von Mises stress in the surrounding breast tissue (see Sect. 2.3.2), whose value depends on the mechanical properties of healthy tissue around the growing tumor. While we use a a linear elasticity framework with a homogeneous Young’s modulus of 3 KPa, previous breast cancer forecasting studies have leveraged a different Young’s modulus in fibroglandular tissue (4 KPa) and adipose tissue (2 KPa) [44–47, 73–75]. Nevertheless, the range of values of the baseline tumor cell diffusivity spans five orders of magnitude (see Table 1), and this parameter was consistently classified as non-influential parameter in all the scenarios of the global sensitivity analysis (see Figs. 4 and 8). Taken together, these observations suggest that a heterogeneous map of Young’s modulus in the range of 2–4 KPa would have very limited impact in the global sensitivity analysis results presented in this work. Nevertheless, heterogeneous mechanical properties can contribute to capture the heterogeneous tumor cell density maps observed in longitudinal MRI measurements during NAC, and hence render superior pointwise tumor forecasts for individual patients [30, 44–47, 73–75]. Thus, while we think that the global sensitivity analysis results are not critically limited by the tissue-box simplification, further studies should investigate the generalization of the reduced model to describe and predict TNBC dynamics during NAC in patient-specific breast geometries featuring heterogeneous mechanical properties.

From a modeling perspective, we only describe breast cancer response to NAC in terms of tumor cell density. Previous clinical-computational studies [44, 46, 74] and our results show that this modeling strategy can capture NAC response ranging from pCR to progressive disease. Nevertheless, this modeling approach may be limited to precisely account for the development of chemoresistance, which is a main cause of chemotherapy failure [117]. To address this limitation, the model can be extended to account for the specific spatiotemporal dynamics of surviving and drug damaged tumor cells [118]. While standard in vivo MRI data offers limited insight into these tumor cell subpopulations, the biophysical mechanisms driving their behavior could be characterized with a global sensitivity analysis informed by in vitro data [58, 118, 119]. Alternatively, if several MRI measurements are collected longitudinally during NAC, our models could be adaptively recalibrated after each new scan (e.g., using data assimilation techniques) and changes in their value could be exploited to identify chemoresistance [120–122]. Furthermore, we assumed that the tumor perfusion map did not change over time in our work. This is a common assumption in prior tumor forecasting studies between consecutive measurements of tumor perfusion via DCE-MRI [44, 45]. Nevertheless, our model could be extended to account for the dynamics of tumor-supported vasculature (i.e., angiogenesis) during NAC [30, 91, 123–128], which could also be the target of therapeutic regimens involving antiangiogenic drugs (e.g., bevacizumab) [2, 12, 91, 129, 130]. This vasculature-coupled model could be further extended by including a computational fluid dynamics representation of blood flow and drug delivery over the breast anatomy [48, 67, 123, 128, 131, 132]. This extension of the model could contribute to achieve a more precise quantification of the drug concentrations in the tumor and healthy tissue, thereby enabling a finer assessment of NAC efficacy and toxicity [48]. Another central modeling assumption is that we leveraged linear elasticity to characterize the TNBC-induced tissue deformation and the effect of local mechanical stress on tumor cell mobility. This modeling strategy has been shown to be a reasonable approximation during the time course of usual NAC regimens [73–75]. Future studies could investigate a mechanical coupling with tumor cell proliferation, which can also be inhibited by local mechanical stress [62, 63, 87, 88, 112] and might thus reduce the efficacy of chemotherapeutic agents [133]. Moreover, a poroelastic formulation of breast tissue would refine the quantification of solid stress and interstitial pressure effects on tumor dynamics and drug distribution, which could render a more accurate description of the spatiotemporal dynamics of local drug availability and treatment effects [63, 87, 128].

The definition of all the model parameters as global scalar quantities over the tissue box constitutes another limitation of our study. This assumption simplifies the construction of the parameter space and the global sensitivity analysis of our model with respect to the use of local parameter maps, which would require formulating a strategy to perform comprehensive and robust spatial sampling of the parameters defined locally. In principle, the use of global or local parameter maps in our model should result in a limited impact in the calculation of global quantities of interest by integrating the tumor cell density field over the computational domain due to spatial averaging (e.g., the tumor volume or total tumor cell count). Thus, we consider that this assumption does not impose a crucial limitation for the results of our global sensitivity analysis. However, the adoption of global parameter values can render a limited representation of the heterogeneous biology that TNBC may exhibit, thereby potentially limiting the agreement between pointwise model predictions and MRI measurements of tumor cell density [30]. To better capture intratumoral heterogeneity, our model could be equipped with local, spatially-resolved parameter maps [44, 45, 47, 73, 74]. In particular, the baseline tumor cell net proliferation rate is consistently identified as a dominant parameter in our work (see Figs. 4 and 8), and the use of tumor cell net proliferation rate maps has yielded highly successful tumor forecasting results in previous studies [44–46, 73–75]. Thus, future tumor forecasting studies using a local formulation of this parameter should evaluate the gain in model performance over a global definition, and could probably observe superior predictive performance at voxelwise level. Alternatively, intratumoral heterogeneity could also be captured by defining parameter subsets or even tumor cell compartments characterizing distinct tumor habitats featuring unique cellularity and perfusion profiles identified via quantitative MRI imaging (e.g., DW-MRI and DCE-MRI) [65]. For example, these tumor habitats could be leveraged to describe normoxic, hypoxic, and necrotic regions of TNBC within our modeling framework [134].

While we adopted a deterministic framework in the sensitivity analysis and modeling study performed in this work, future studies could extend our analysis by leveraging a Bayesian framework. This approach can further account for the uncertainties in the different sources of the parameter estimates as well as in the model outcomes (e.g., $V_{T}$ and $N_{T}$) [30, 32, 112, 135]. Moreover, a Bayesian framework would allow for investigating alternative model versions within a model selection study [30, 112]. These alternative models may include all or a subset of the dominant parameters identified in our sensitivity analysis, recruit some non-influential parameters to boost model performance (e.g, parameters describing cylophosphamide effects and their synergies with doxorubicin; see Fig. 4), and feature some of the extensions of the model formulation discussed above. Additionally, the computer simulations in our work rely on isogeometric analysis, which is a recent generalization of classic finite element analysis [107], and the average time of simulation for each parameter combination ranged from approximately 20–40 min. While these computational times are viable in an academic setting to investigate the model dynamics, the clinical implementation of our model would require the development of advanced isogeometric methods to reduce the computational time required to run all the simulations that are necessary to obtain a personalized prediction of NAC response. For example, after the preprocessing of the imaging data collected for a new patient, a deterministic implementation of personalized tumor forecasting would require a reduced number of simulations for model calibration and a final simulation to predict NAC response [30, 47]. However, the computational cost can become prohibitive for a Bayesian implementation for uncertainty quantification and model selection because these techniques require a large number of model simulations to construct probability distributions [30, 112, 135]. To address these computational limitations for the clinical implementation of our model, reduced order modeling and scientific machine learning can be used to accelerate computer simulations of tumor growth models [136, 137].

In the future, we plan to investigate the performance of the reduced model identified in this study on recapitulating TNBC response during NAC using in vivo quantitative MRI data of individual patients in a diverse cohort (e.g., different tumor locations in the breast, sizes, and perfusion profiles). We further plan to assess whether model forecasts of TNBC response obtained by informing the model with just two longitudinal MRI datasets acquired early in the course of NAC (e.g., at baseline and after one or two cycles) suffice to accurately predict therapeutic outcome at the conclusion of the prescribed NAC regimen for each patient [44, 45, 47]. In particular, these two studies can quantify the ability of the reduced model to capture changes in tumor volume and total cell counts for individual patients, as well as the extent to which this model can capture tumor heterogeneity as observed in MRI measurements. This capability would enable the treating oncologist to identify non-responding patients early in the course of NAC, who could benefit from switching to other therapeutics, as well as exceptional responders, who could benefit from de-escalation or even avoidance of the ensuing breast surgery [10, 11, 13–16]. Additionally, while clinical studies cannot evaluate the complex landscape of potential drug combinations, dosages, and scheduling strategies (e.g., timing and order of drug delivery), a validated mechanistic model of TNBC response to NAC supported by a robust parameter space characterizing potential baseline tumor dynamics and combined drug effects would constitute a powerful technology to rigorously and systematically design optimal therapeutic plans for individual patients [30, 45, 48, 130]. Hence, this technology could be cast as a digital twin of the patient’s tumor that assimilates upcoming imaging or clinical data as they become available to update the prediction of NAC outcome [46, 135, 138, 139]. Then, this digital twin would provide the treating oncologist with a comprehensive picture of observed and expected tumor dynamics during the prescribed NAC regimen and potential alternative treatments, thereby supporting optimal clinical decision-making for each individual patient.

## Conclusion

We have presented a new mechanistic model which extends an established mechanically-coupled, drug-informed formulation of breast cancer response to NAC with an explicit term representing the pharmacokinetics and pharmacodynamics of the drugs in the NAC regimen. We further investigated the mechanisms in the model that drive TNBC response to two usual NAC regimens (doxorubicin plus cyclophosphamide, and paclitaxel plus carboplatin) by performing a global variance-based sensitivity analysis in two distinct scenarios: a well-perfused and poorly perfused tumor, which are defined upon two corresponding patient-specific in vivo MRI data. To this end, we constructed the parameter space of our model by integrating in vitro measurements of TNBC cell response to the NAC drugs of interest via high-throughput automated microscopy, and in silico estimates of model parameters from prior MRI-informed tumor forecasting studies in TNBC patients receiving the NAC regimens under investigation. Our sensitivity analysis identified the drug-induced changes in tumor cell net proliferation caused by doxorubicin, paclitaxel, and carboplatin as the main drivers of TNBC response to the two NAC regimens considered herein. Out of the $n_ {p}=15$ parameters included in the sensitivity analysis, our results show that these driving model mechanisms are ultimately regulated by only three parameters in the doxorubicin and cyclophosphamide regimen (the baseline tumor cell net proliferation, the normalized maximal effect of doxorubicin, and the normalized maximal concentration of doxorubicin) and only five parameters in the paclitaxel and carboplatin regimen (the baseline tumor cell net proliferation, the normalized maximal effect of both drugs, and the normalized maximal concentration of both drugs). Additionally, we demonstrated that a reduced version of our model that is solely driven by the dominant parameters can be leveraged as a valid surrogate of our original model, since both models achieve a high level of qualitative and quantitative agreement while also enabling the simulation of the diverse spectrum of NAC outcomes observed in the clinic (i.e, from pCR to progressive disease). Thus, we posit that the reduced model is an excellent candidate to compute personalized MRI-informed predictions of NAC outcome, and we further envision that this model could ultimately support a personalized digital twin to optimize clinical decision-making during NAC for each individual TNBC patient.

## Supplementary Information

Below is the link to the electronic supplementary material.

Supplementary file 1 (pdf 272 KB)

## Data Availability

The anonymized MRI measurements used in this work are available at Zenodo (URL: https://doi.org/10.5281/zenodo.7971351), along with a collection of MATLAB scripts to perform the following tasks: preprocessing of the MRI data (as explained in Sect. S3 of the Supplementary Methods), generation of the random parameter samples for the global sensitivity analysis, calculation of the total-effects indices, and postprocessing analyses on the results of the global sensitivity analysis. The full anonymized MRI dataset is not currently available in open access, but it can be available upon request from the authors of Ref. [45]. Additionally, the experimental data of tumor cell counts and extracted drug-induced proliferation changes are available at Thunor (URL: https://thunor.app.vanderbilt.edu/dataset/15). These data along with the experimentally determined MuSyC model parameters can be visualized in the online MuSyC application (URL: https://musyc.app.vanderbilt.edu/accounts/login/?next=/).
