# Supplementary material for: A global sensitivity analysis of a mechanistic model of neoadjuvant chemotherapy for triple negative breast cancer constrained by in vitro and in vivo imaging data
Source: Eng Comput. 2023 Aug 7;40(3):1469–99. doi: 10.1007/s00366-023-01873-0 (PMC11607094; doi:10.1007/s00366-023-01873-0)
Supplement: Supplementary file 1 — (pdf 272 KB) [file 366_2023_1873_MOESM1_ESM.pdf]

Supplementary Information

A global sensitivity analysis of a mechanistic model of neoadjuvant chemotherapy for triple negative breast cancer constrained by *in vitro* and *in vivo* imaging data

Guillermo Lorenzo[1,2,*], Angela M. Jarrett[2,3], Christian D. Meyer[4,5], Julie C. DiCarlo[2,3,6], John Virostko[2,3,7,8], Vito Quaranta[4,5,9,10], Darren R. Tyson[4,9,10], Thomas E. Yankeelov[2,3,7,8,11,12]

[1]Department of Civil Engineering and Architecture, University of Pavia, Pavia, Italy

[2]Oden Institute for Computational Engineering and Sciences, The University of Texas at Austin, Austin, TX, USA

[3]Livestrong Cancer Institutes, Dell Medical School, The University of Texas at Austin, Austin, TX, USA

[4]Center for Cancer Systems Biology at Vanderbilt, Vanderbilt University, Nashville, TN, USA

[5]Duet BioSystems, Inc., Nashville, TN, USA

[6]Biomedical Imaging Center, The University of Texas at Austin, Austin, TX, USA

[7]Department of Diagnostic Medicine, The University of Texas at Austin, Austin, Austin, TX, USA

[8]Department of Oncology, The University of Texas at Austin, Austin, Austin, TX, USA

[9]Department of Biochemistry, Vanderbilt University, Nashville, TN, USA

[10]Department of Pharmacology, Vanderbilt University School of Medicine, Nashville, TN, USA

[11]Department of Biomedical Engineering, The University of Texas at Austin, Austin, TX, USA

[12]Department of Imaging Physics, The University of Texas MD Anderson Cancer Center, Houston, TX, USA

*Corresponding author

Department of Civil Engineering and Architecture

University of Pavia

Via Ferrata 3, 27100, Pavia, Italy

Email: guillermo.lorenzo@unipv.it, guillermo.lorenzo@utexas.edu

Supplementary Methods

S1. MRI data acquisition and preprocessing

The two magnetic resonance imaging (MRI) datasets leveraged in this work to construct the well-perfused and the poorly-perfused scenarios were obtained from the dataset used in our previous study on breast cancer forecasting presented in Ref. [S1]. In the following, we provide an extended description of the MRI acquisition protocol and ensuing preprocessing that were used in that study to support the outline presented in the main text of the article. The interested reader is referred to Refs. [S1, S2] for further information.

S1.1. MRI data acquisition

Two imaging facilities provided the MRI datasets for the study carried out in Ref. [S1]: an outpatient imaging facility and a regional hospital. Both centers routinely perform breast MRI. Siemens 3T Skyra scanners (Erlangen, Germany) equipped with either an 8- or 16-channel receive double-breast coil (Sentinelle, Invivo, Gainesville, Florida, USA) were leveraged to collect the MRI acquisitions. The patient data were collected following an institutional review board-approved and HIPAA-compliant protocol, where patients provided informed consent to participate in a longitudinal MRI study throughout the course of their standard-of-care neoadjuvant therapy. All MRI data was collected in the sagittal plane over a 256 × 256 mm^2 field of view.

Diffusion-weighted MRI (DW-MRI). A monopolar, single-shot spin echo, echo planar imaging sequence in a diagonal diffusion-encoding direction was used to collect the DW-MRI data. Six acquisitions were averaged for b-values of 0 and 200 s/mm^2 and 18 acquisitions were averaged for a b-value of 800 s/mm2, thereby allowing for approximately equal signal-to-noise ratios at all b-values. DW-MRI data were collected over 10 slices, with 5 mm thickness, and no slice gap. Spectrally selective adiabatic inversion recovery (SPAIR) fat suppression was implemented for a total scan time of 1 minute 39 seconds. Additional acquisition parameters for the DW-MRI data were as follows: repetition time/echo time (*TR/TE*) = 3000/52 ms, flip angle 90°, acquisition matrix of 128 × 128, and a GeneRalized Autocalibrating Partial Parallel Acquisition (GRAPPA) acceleration of 2.

High-resolution T_1-weighted MRI (T1W-MRI). A high-resolution T_1-weighted, 3D gradient-echo, FLASH (fast low angle shot) MRI dataset was acquired using the following parameters: *TR/TE* = 5.3/2.3 milliseconds, flip angle = 10°, acquisition matrix = 256 × 256, slice thickness = 1 mm, GRAPPA acceleration of 2, and SPAIR (Spectral Selection Attenuated Inversion Recovery) fat suppression. This anatomical image was acquired in 3 minutes and 11 seconds.

Dynamic contrast-enhanced MRI (DCE-MRI). The DCE-MRI protocol was based on a T_1-weighted, VIBE (Volumetric Interpolated Breath-hold Examination; although no breathholding was employed in these studies) acquisition with *TR/TE* = 7.02/4.6 ms, flip angle of 6°, acquisition matrix of 192 × 192, with 10 slices of 5 mm thickness each with 1.3 × 1.3 mm^2 resolution in-plane, and a GRAPPA acceleration factor of 2. This

protocol enabled a temporal resolution of 7.27 seconds per volume for 1 minute prior to and 6 minutes post administration of a gadolinium-based contrast agent (Multihance (Bracco, Monroe Township, NJ) or Gadovist (Bayer, Leverkusen, Germany)) using a power injector followed by a saline flush.

***T_1* mapping.** A T_1-weighted 3D spoiled gradient echo image was acquired with variable flip angles spanning 2° to 20° in 2° increments to calculate the longitudinal relaxation time, T_1. Acquisition parameters included: *TR/TE* = 7.9/2.4 milliseconds, acquisition matrix = 192 × 192, 10 slices of 5 mm thickness each with 1.3 × 1.3 mm^2 resolution in-plane, and a GRAPPA acceleration factor of 3. The total acquisition time was 50 seconds.

***B_1* mapping.** A Siemens TurboFLASH sequence was utilized to map the B_1 field to correct for transmit inhomogeneity and correct the T_1 map. The acquisition leveraged the following parameters: *TR/TE* = 8680/2 milliseconds, flip angle = 8°, acquisition matrix = 96 × 96, and slice thickness = 5 mm with 2.7 × 2.7 mm^2 resolution in-plane. Due to the inclusion of a slice gap in the B_1 mapping protocol, two acquisitions were performed to cover the same slices as the aforementioned MRI measurements. The total acquisition time was 34 seconds.

S1.2. MRI data preprocessing

The preprocessing of the longitudinal MRI datasets for each patient included intra- and inter-scan registration, segmentation, and calculation of parameter fields and other quantities of interest to inform a mechanistic model of breast cancer growth and response to neoadjuvant therapy (see Refs. [S1, S2] for further detail).

Registration. For each patient, the intra-scan registration consisted of a rigid registration algorithm, whereby the B1, T1W, and DW images were aligned to the DCE images collected during the same MRI session. The images that were acquired at a different resolution compared to the DCE images were resampled using a nearest neighbor approach *via* function *interp3* from MATLAB (MathWorks, Natick, MA). The rigid algorithm used for the intra-scan registration was implemented using MATLAB's function *imregister*. Then, the inter-scan registration aligned all MRI datasets across time to a common space for each patient by leveraging a non-rigid registration algorithm with a constraint that preserves the tumor volumes at each time point [S3]. This registration employs an adaptive basis algorithm performed using the software Elastix [S4, S5].

Segmentation. The tumor region of interest was segmented on DCE-MRI data at each time point by utilizing a fuzzy c-means-based clustering algorithm [S6]. The breast tissue was segmented into fibroglandular and adipose tissue. Towards this end, MATLAB's *adapthisteq* function was first applied to enhance the post inter-scan registered DCE images by using a contrast-limited adaptive histogram equalization algorithm. Then, fibroglandular and adipose tissue masks were segmented using a *k*-means clustering algorithm.

Vasculature. The standard Kety-Tofts model [S7] was fit to the DCE-MRI data from each voxel within the tumor using a population-averaged arterial input function of contrast concentration in plasma that was established for the population in Ref. [S1] according to the method presented in Ref. [S8]. The voxels for which the Kety-Tofts model fit did not converge or converged to non-physical values based on the spoiled gradient echo signal equation were removed. As a result, the fitting of the Kety-Tofts model provided spatial maps of the volume transfer constant from the plasma space to the tissue space, and maps of the volume fraction of the extravascular extracellular space. Additionally, a normalized map of the blood volume was calculated by calculating the area under the dynamic curve (AUC) of the baseline-subtracted time course for each voxel, and then normalizing by the maximum AUC value from the whole tumor region of interest. The resulting normalized blood volume map can be leveraged to inform the term describing the spatiotemporal dynamics of drug concentration in organ-scale mechanistic models of breast cancer growth and treatment response [S1, S9].

Tumor cell density. The apparent diffusion coefficient (ADC) maps were calculated from the DW-MRI data at each timepoint by using standard methods [S10]. Then, the ADC value for each voxel within the tumor region of interest was converted to an estimate for the number of tumor cells per *via* an established formulation that has been successfully leveraged in tumor forecasting studies [S1, S9, S11-S13].

S2. Selection of the well-perfused and poorly-perfused tumor datasets

First, we took the MRI datasets from the patients who received the two NAC regimens considered in our work (i.e., doxorubicin plus cyclophosphamide, and paclitaxel plus carboplatin) at one of the medical institutions participating in Ref. [S1]. This resulted in an initial group of 13 patients. A first qualitative analysis of the imaging data showed that one of the datasets had DW-MRI artifacts caused by a silicone implant, so that patient was dropped (i.e., n = 12). Then, we classified the patients into two balanced subgroups of six patients each according to the median of the normalized map of blood volume over the tumor. This map was used in the mathematical model proposed in Ref. [S1] as part of the formulation to describe the drug effects on breast tumor dynamics (see Section S1.2 above). The subgroup of breast cancer cases with higher median normalized blood volume over the tumor region of interest was termed "well-perfused tumors", and the other group was characterized as the "poorly-perfused tumors". Finally, we chose one tumor from either group, such that their corresponding bounding box had no more than 1600 in-plane voxels to ensure the computational tractability of the elevated number of model simulations in the global variance-based sensitivity analysis of our study (see Section 2.5 in the main text). Supplementary Figure S1 shows the boxplots of the normalized blood volume for the 12 patients considered in this analysis, also indicating the two that were ultimately used to construct the well-perfused and poorly-perfused tumor scenarios in our work.

S3. Generation of isogeometric meshes for the tumor scenarios from MRI data

As outlined in Section 2.4.1 of the main text, we discretize our biomechanistic model of breast cancer response to neoadjuvant chemotherapy (NAC) in space by leveraging a standard isogeometric Bubnov-Galerkin method relying on a 3D C^1 quadratic B-spline space [S14-S18]. The construction of the isogeometric meshes for the well-perfused and poorly-perfused tumor scenarios consisted of upsampling the original voxel grid of the corresponding tissue boxes (see Fig.1 and Section 2.1 in the main text) to achieve sufficient numerical accuracy in the computer simulations of our model. This procedure was performed in two steps: first we upsampled the out-of-plane resolution (i.e., the slice thickness) by a factor of 4 to approximately match the in-plane resolution, and then we upsampled the resulting voxel grid by a factor 2 to obtain the same resolution as the target isogeometric element mesh. The original tissue box of the well-perfused tumor scenario consisted of 33×33×7 voxels with size 1.33×1.33×5 mm, and it was resampled to define an isogeometric mesh of 66×66×56 elements with size 0.67×0.67×0.63 mm. Likewise, the original tissue box of the poorly-perfused scenario consisted of 34×45×7 voxels with size 1.33×1.33×5 mm, and it was refined to define an isogeometric mesh of 68×90×56 elements with size 0.67×0.67×0.63 mm.

The imaging data in the resampled tissue boxes (e.g., tumor cell density, perfusion) was further upsampled by a factor of 3, and the resulting values were assigned to the Gauss quadrature points within each element of the isogeometric mesh (see Section 2.4.3 in the main text) [S14-S17]. In particular, the imaging measurements of tumor cell density were L^2-projected over the spline space to set the initial condition for the breast tumor dynamics in Eq. (1) in Section 2.3.1 of the main text [S16-S18]. To obtain a smooth spline representation of the initial tumor cell density for numerical accuracy of the solvers in Section 2.4.3 of the main text, Gauss filtering provided by *imgaussfilt3* in MATLAB 2021a (The Mathworks, Natick, MA) was leveraged after the first upsampling step over the tumor ROI border. This method was applied to smooth the transition between the imaging measurements of tumor cell density and the neighboring healthy tissue (i.e., where $N(\mathbf{x},t) = 0$; see Fig. 1 in the main text) without altering the intratumoral cell density values. Hence, this procedure facilitated an initial approximation of the diffusive front that ultimately emanates from Eq. (1) in the main text (see Section 2.3.1).

References

[S1] Jarrett, A. M., Hormuth II, D. A., Wu, C., Kazerouni, A. S., Ekrut, D. A., Virostko, J., *et al.* (2020). Evaluating patient-specific neoadjuvant regimens for breast cancer via a mathematical model constrained by quantitative magnetic resonance imaging data. *Neoplasia*, 22(12), 820-830.

[S2] Jarrett, A. M., Kazerouni, A. S., Wu, C., Virostko, J., Sorace, A. G., DiCarlo, J. C., *et al.* (2021). Quantitative magnetic resonance imaging and tumor forecasting of breast cancer patients in the community setting. *Nature Protocols*, 16(11), 5309-5338.

[S3] Li, X., Dawant, B. M., Welch, E. B., Chakravarthy, A. B., Xu, L., Mayer, I., *et al.* (2010). Validation of an algorithm for the nonrigid registration of longitudinal breast MR images using realistic phantoms. *Medical Physics*, 37(6Part1), 2541-2552.

[S4] Klein, S., Staring, M., Murphy, K., Viergever, M. A., and Pluim, J. P. (2009). Elastix: a toolbox for intensity-based medical image registration. *IEEE Transactions on Medical Imaging*, 29(1), 196-205.

[S5] Shamonin, D. P., Bron, E. E., Lelieveldt, B. P., Smits, M., Klein, S., Staring, M., and Alzheimer's Disease Neuroimaging Initiative. (2014). Fast parallel image registration on CPU and GPU for diagnostic classification of Alzheimer's disease. *Frontiers in Neuroinformatics*, 7, 50.

[S6] Wu, C., Pineda, F., Hormuth, D. A., Karczmar, G. S., and Yankeelov, T. E. (2019). Quantitative analysis of vascular properties derived from ultrafast DCE-MRI to discriminate malignant and benign breast tumors. *Magnetic Resonance in Medicine*, 81(3), 2147-2160.

[S7] Yankeelov, T. E., and Gore, J. C. (2007). Dynamic contrast enhanced magnetic resonance imaging in oncology: theory, data acquisition, analysis, and examples. *Current Medical Imaging*, 3(2), 91-107.

[S8] Li, X., Welch, E. B., Arlinghaus, L. R., Chakravarthy, A. B., Xu, L., Farley, J., *et al.* (2011). A novel AIF tracking method and comparison of DCE-MRI parameters using individual and population-based AIFs in human breast cancer. *Physics in Medicine & Biology*, 56(17), 5753.

[S9] Jarrett, A. M., Hormuth, D. A., Barnes, S. L., Feng, X., Huang, W., and Yankeelov, T. E. (2018). Incorporating drug delivery into an imaging-driven, mechanics-coupled reaction diffusion model for predicting the response of breast cancer to neoadjuvant chemotherapy: theory and preliminary clinical results. *Physics in Medicine & Biology*, 63(10), 105015.

[S10] Whisenant, J. G., Ayers, G. D., Loveless, M. E., Barnes, S. L., Colvin, D. C., and Yankeelov, T. E. (2014). Assessing reproducibility of diffusion-weighted magnetic resonance imaging studies in a murine model of HER2+ breast cancer. *Magnetic Resonance Imaging*, 32(3), 245-249.

[S11] Atuegwu, N. C., Arlinghaus, L. R., Li, X., Chakravarthy, A. B., Abramson, V. G., Sanders, M. E., and Yankeelov, T. E. (2013). Parameterizing the logistic model of tumor growth by DW-MRI and DCE-MRI data to predict treatment response and changes in breast cancer cellularity during neoadjuvant chemotherapy. *Translational Oncology*, 6(3), 256-264.

[S12] Weis, J. A., Miga, M. I., Arlinghaus, L. R., Li, X., Chakravarthy, A. B., Abramson, V., *et al.* (2013). A mechanically coupled reaction–diffusion model for predicting the response of breast tumors to neoadjuvant chemotherapy. *Physics in Medicine & Biology*, 58(17), 5851.

[S13] Weis, J. A., Miga, M. I., Arlinghaus, L. R., Li, X., Abramson, V., Chakravarthy, A. B., *et al.* (2015). Predicting the Response of Breast Cancer to Neoadjuvant Therapy Using a Mechanically Coupled Reaction–Diffusion Model. *Cancer Research*, 75(22), 4697-4707.

[S14] Cottrell, J.A., Hughes, T.J.R., and Bazilevs, Y. *Isogeometric analysis: toward integration of CAD and FEA*. John Wiley & Sons, 2009.

[S15] Lorenzo, G., Hormuth II, D.A., Jarrett, A.M., Lima, E.A., Subramanian, S., Biros, G., Oden, J.T., Hughes, T.J., and Yankeelov, T.E. Quantitative in vivo imaging to enable tumor forecasting and treatment optimization. In: Balaz, I. and Adamatzky, A., editors, *Cancer, Complexity, Computation*. Springer, 2023.

[S16] Lorenzo, G., Hughes, T.J.R., Dominguez-Frojan, P., Reali, A., and Gomez, H. Computer simulations suggest that prostate enlargement due to benign prostatic hyperplasia mechanically impedes prostate cancer growth. *Proceedings of the National Academy of Sciences of the U.S.A.*, 116(4):1152–1161, 2019.

[S17] Lorenzo, G., Hughes, T., Reali, A., and Gomez, H. A numerical simulation study of the dual role of 5alpha-reductase inhibitors on tumor growth in prostates enlarged by benign prostatic hyperplasia via stress relaxation and apoptosis upregulation. *Computer Methods in Applied Mechanics and Engineering*, 362:112843, 2020.

[S18] Colli, P., Gomez, H., Lorenzo, G., Marinoschi, G., Reali, A., and Rocca, E. Mathematical analysis and simulation study of a phase-field model of prostate cancer growth with chemotherapy and antiangiogenic therapy effects. *Mathematical Models and Methods in Applied Sciences*, 30(07):1253–1295, 2020.

Supplementary Figures

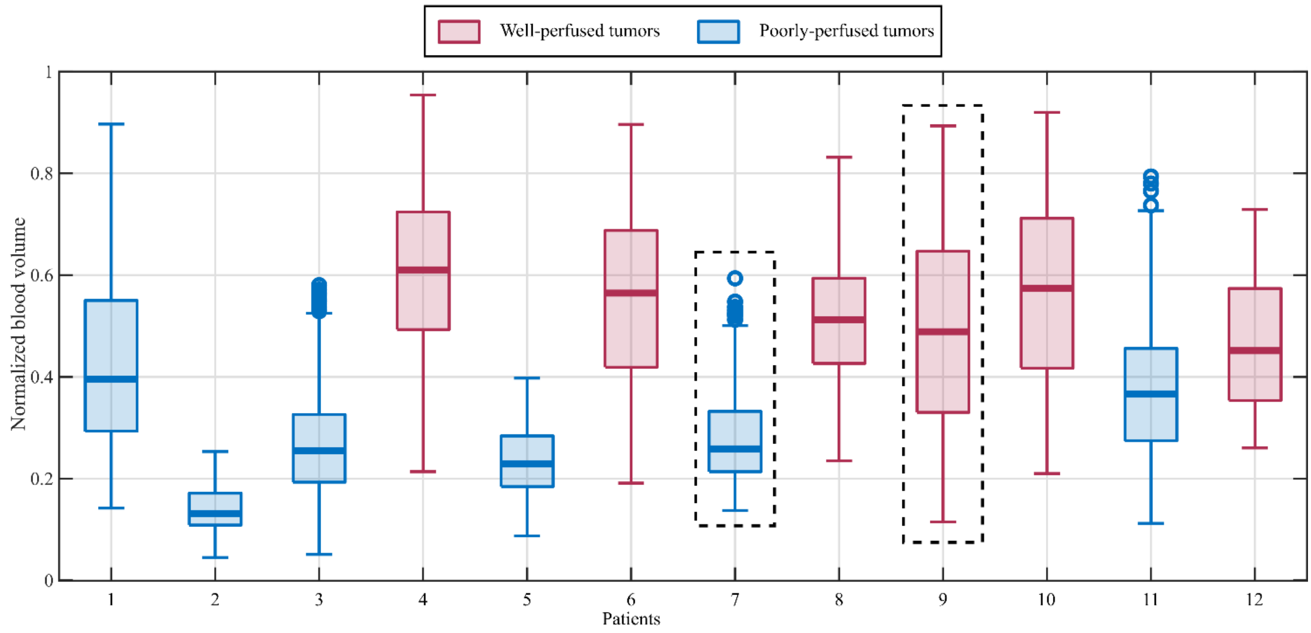

Supplementary Figure S1. Distribution of normalized blood volume over the tumor region of interest across 12 patients from one of the medical institutions participating in Ref. [S1]. The patients were ranked based on their median normalized blood volume over the tumor and classified in two balanced groups of 6 patients each. Hence, tumors exhibiting higher normalized blood volume values were termed "well-perfused", and those with lower values for this parameter were called "poorly-perfused". In this figure, the boxplots of normalized blood volume values for each type of tumor are shown in red and blue, respectively. Outliers are represented as hollow circles in matching colors. The well-perfused and poorly-perfused tumor used in our global variance-based sensitivity analysis are indicated with a dashed box.

Supplementary Tables

Parameters [units]	TNBC cell line			
	HCC1143	SUM149	MDAMB231	MDAMB468
ρ_0 [h^{-1}]	$2.61\cdot10^{-2}$ [$2.52\cdot10^{-2}$, $2.61\cdot10^{-2}$]	$2.23\cdot10^{-2}$ [$2.14\cdot10^{-2}$, $2.23\cdot10^{-2}$]	$2.96\cdot10^{-2}$ [$2.90\cdot10^{-2}$, $3.02\cdot10^{-2}$]	$2.24\cdot10^{-2}$ [$2.16\cdot10^{-2}$, $2.24\cdot10^{-2}$]
$\hat{E}_1$ [-]	0.15 [0.15, 0.16]	0.11 [0.10, 0.11]	-0.02 [-0.03, 0.01]	0.02 [0.00, 0.08]
$\hat{E}_2$ [-]	-0.15 [-0.20, -0.05]	-0.19 [-0.20, -0.05]	-0.20 [-0.21, -0.13]	-0.32 [-0.33, -0.22]
C_1 [M]	$7.04\cdot10^{-9}$ [$5.74\cdot10^{-9}$, $7.04\cdot10^{-9}$]	$9.60\cdot10^{-10}$ [$7.04\cdot10^{-10}$, $9.60\cdot10^{-10}$]	$1.39\cdot10^{-8}$ [$1.27\cdot10^{-8}$, $1.45\cdot10^{-8}$]	$8.84\cdot10^{-9}$ [$7.38\cdot10^{-9}$, $1.31\cdot10^{-8}$]
C_2 [M]	$6.52\cdot10^{-5}$ [$7.52\cdot10^{-6}$, $1.50\cdot10^{-3}$]	$2.16\cdot10^{-6}$ [$1.40\cdot10^{-6}$, $3.11\cdot10^{-6}$]	$2.74\cdot10^{-6}$ [$2.16\cdot10^{-6}$, $3.06\cdot10^{-6}$]	$3.19\cdot10^{-7}$ [$1.35\cdot10^{-7}$, $4.87\cdot10^{-7}$]
h_1 [-]	2.05 [1.53, 2.68]	1.05 [1.05, 1.57]	2.64 [2.08, 3.13]	4.79 [2.79, 24.15]
h_2 [-]	0.63 [0.51, 1.24]	1.22 [0.92, 2.70]	1.21 [1.21, 1.56]	0.47 [0.42, 0.65]
α_1 [-]	$5.44\cdot10^{-3}$ [$2.54\cdot10^{-3}$, $2.03\cdot10^{2}$]	$3.82\cdot10^{-1}$ [$6.85\cdot10^{-2}$, 1.57]	$1.63\cdot10^{-1}$ [$7.53\cdot10^{-2}$, 2.29]	$4.17\cdot10^{-4}$ [$1.00\cdot10^{-4}$, $1.07\cdot10^{3}$]
α_2 [-]	$3.61\cdot10^{-4}$ [$1.00\cdot10^{-4}$, 7.62]	$1.00\cdot10^{-4}$ [$1.00\cdot10^{-4}$, $3.93\cdot10^{1}$]	3.44 [2.24, 5.05]	2.25 [1.06, 4.30]
β [-]	$-1.19\cdot10^{-1}$ [$-1.80\cdot10^{-1}$, $-1.53\cdot10^{-3}$]	$1.70\cdot10^{-2}$ [$-7.62\cdot10^{-3}$, $2.94\cdot10^{-1}$]	$-4.51\cdot10^{-2}$ [$-6.09\cdot10^{-2}$, $2.71\cdot10^{-2}$]	$-1.68\cdot10^{-1}$ [$-1.95\cdot10^{-1}$, $-8.12\cdot10^{-2}$]

Supplementary Table S1. Experimental measurements of the MuSyC pharmacodynamic parameters in four TNBC cell lines treated with doxorubicin and perfosfamide (subindices 1 and 2, respectively), which was used *in lieu* of the pro-drug cyclophosphamide. For each parameter, this table reports the average value and corresponding 95% confidence interval obtained from analyzing within the MuSyC framework a collection of high-throughput, time-resolved automated microscopy datasets assessing the response of TNBC cells from each of the four lines to varying concentrations of doxorubicin and perfosfamide delivered in combination. Section 2.2 in the main text provides further detail about the experimental setup. The normalization of $\hat{E}_1$ and $\hat{E}_2$ was calculated *a posteriori* with respect to the average value of ρ_0 reported in this table for each cell line.

Parameters [units]	TNBC cell line			
	HCC1143	SUM149	MDAMB231	MDAMB468
ρ_0 [h^{-1}]	$2.50 \cdot 10^{-2}$<br>[$2.39 \cdot 10^{-2}$, $2.57 \cdot 10^{-2}$]	$2.20 \cdot 10^{-2}$<br>[$2.11 \cdot 10^{-2}$, $2.20 \cdot 10^{-2}$]	$3.02 \cdot 10^{-2}$<br>[$2.95 \cdot 10^{-2}$, $3.02 \cdot 10^{-2}$]	$2.24 \cdot 10^{-2}$<br>[$2.19 \cdot 10^{-2}$, $2.24 \cdot 10^{-2}$]
$\hat{E}_1$ [-]	-0.10<br>[-0.12, -0.02]	-0.04<br>[-0.05, 0.02]	-0.04<br>[-0.07, 0.02]	-0.39<br>[-0.41, -0.37]
$\hat{E}_2$ [-]	0.51<br>[0.48, 0.58]	-0.03<br>[-0.06, 0.01]	0.35<br>[0.32, 0.40]	-0.51<br>[-0.53, -0.40]
C_1 [M]	$1.38 \cdot 10^{-9}$<br>[$1.07 \cdot 10^{-9}$, $1.84 \cdot 10^{-9}$]	$3.19 \cdot 10^{-9}$<br>[$2.54 \cdot 10^{-9}$, $3.90 \cdot 10^{-9}$]	$1.78 \cdot 10^{-9}$<br>[$1.58 \cdot 10^{-9}$, $2.03 \cdot 10^{-9}$]	$2.11 \cdot 10^{-9}$<br>[$2.03 \cdot 10^{-9}$, $2.22 \cdot 10^{-9}$]
C_2 [M]	$5.66 \cdot 10^{-7}$<br>[$7.00 \cdot 10^{-8}$, $5.66 \cdot 10^{-7}$]	$5.66 \cdot 10^{-7}$<br>[$3.85 \cdot 10^{-7}$, $5.66 \cdot 10^{-7}$]	$5.66 \cdot 10^{-7}$<br>[$2.21 \cdot 10^{-7}$, $5.66 \cdot 10^{-7}$]	$1.89 \cdot 10^{-5}$<br>[$8.48 \cdot 10^{-6}$, $3.39 \cdot 10^{-5}$]
h_1 [-]	1.62<br>[1.20, 2.64]	2.71<br>[1.95, 4.54]	2.40<br>[1.78, 3.56]	4.11<br>[3.49, 5.18]
h_2 [-]	0.59<br>[0.32, 2.98]	0.61<br>[0.42, 0.83]	0.48<br>[0.35, 1.41]	0.82<br>[0.69, 1.11]
α_1 [-]	$3.90 \cdot 10^{-3}$<br>[$1.00 \cdot 10^{-4}$, $1.00 \cdot 10^{4}$]	$1.00 \cdot 10^{-4}$<br>[$1.00 \cdot 10^{-4}$, $4.30 \cdot 10^{1}$]	$1.00 \cdot 10^{4}$<br>[$1.19 \cdot 10^{-1}$, $1.00 \cdot 10^{4}$]	1.01<br>[$5.15 \cdot 10^{-4}$, $1.00 \cdot 10^{4}$]
α_2 [-]	$2.96 \cdot 10^{-1}$<br>[$1.00 \cdot 10^{-4}$, $8.36 \cdot 10^{-1}$]	$5.29 \cdot 10^{-4}$<br>[$1.00 \cdot 10^{-4}$, 2.24]	$9.30 \cdot 10^{-4}$<br>[$1.00 \cdot 10^{-4}$, $8.08 \cdot 10^{-1}$]	1.26<br>[$2.57 \cdot 10^{-4}$, 1.90]
β [-]	$-4.90 \cdot 10^{-3}$<br>[$-2.34 \cdot 10^{-2}$, $1.79 \cdot 10^{-1}$]	$1.97 \cdot 10^{-3}$<br>[$-4.43 \cdot 10^{-4}$, $7.96 \cdot 10^{-2}$]	$5.86 \cdot 10^{-2}$<br>[$2.93 \cdot 10^{-2}$, $1.60 \cdot 10^{-1}$]	$-8.45 \cdot 10^{-2}$<br>[$-1.03 \cdot 10^{-1}$, $-6.97 \cdot 10^{-5}$]

Supplementary Table S2. Experimental measurements of the MuSyC pharmacodynamic parameters in four TNBC cell lines treated with paclitaxel and carboplatin (subindices 1 and 2, respectively). For each parameter, this table reports the average value and corresponding 95% confidence interval obtained from analyzing within the MuSyC framework a collection of high-throughput, time-resolved automated microscopy datasets assessing the response of TNBC cells from each of the four lines to varying concentrations of paclitaxel and carboplatin delivered in combination (see Section 2.2 in the main text). Section 2.2 in the main text provides further detail about the experimental setup. The normalization of $\hat{E}_1$ and $\hat{E}_2$ was calculated *a posteriori* with respect to the average value of ρ_0 reported in this table for each cell line.

Parameters [units]	Fixed values in reduced model
D_0 [mm^{2}d^{-1}]	$3.10 \cdot 10^{-4}$
$\hat{E}_2$ [-]	-2.25
C_1 [M]	$5.37 \cdot 10^{-9}$
C_2 [M]	$3.31 \cdot 10^{-6}$
$\hat{d}_{m,2}$ [-]	70.71
γ_1 [d^{-1}]	0.40
γ_2 [d^{-1}]	2.89
h_1 [-]	2.29
h_2 [-]	2.20
α_1 [-]	$1.95 \cdot 10^{-2}$
α_2 [-]	$2.29 \cdot 10^{-2}$
β [-]	-0.08

Supplementary Table S3. Fixed values of the non-influential parameters for the reduced version of our biomechanistic model of breast cancer response to NAC based on doxorubicin and cyclophosphamide (subindices 1 and 2, respectively). These non-influential parameters were identified in the sensitivity analysis of the model to this particular NAC regimen, and the values reported in this table were chosen within the ranges reported in Table 1 in the main text.

Parameters [units]	Fixed values in reduced model
D_0 [mm^{2}d^{-1}]	$3.10\cdot10^{-4}$
C_1 [M]	$5.37\cdot10^{-9}$
C_2 [M]	$3.31\cdot10^{-6}$
γ_1 [d^{-1}]	0.40
γ_2 [d^{-1}]	2.89
h_1 [-]	2.29
h_2 [-]	2.20
α_1 [-]	$1.95\cdot10^{-2}$
α_2 [-]	$2.29\cdot10^{-2}$
β [-]	-0.08

Supplementary Table S4. Fixed values of the non-influential parameters for the reduced version of our biomechanistic model of breast cancer response to NAC based on paclitaxel and carboplatin (subindices 1 and 2, respectively). These non-influential parameters were identified in the sensitivity analysis of the model to this particular NAC regimen, and the values reported in this table were chosen within the ranges reported in Table 1 in the main text.

Parameters [units]	Model simulations in Figure 7			
	Panel A	Panel B	Panel C	Panel D
D_0 [mm^2d^{-1}]	$3.10 \cdot 10^{-4}$			
ρ_0 [d^{-1}]	$2.50 \cdot 10^{-2}$			
$\hat{E}_1$ [-]	-4.00	-2.00	-2.50	-1.50
$\hat{E}_2$ [-]	-2.25			
C_1 [M]	$5.37 \cdot 10^{-9}$			
C_2 [M]	$3.31 \cdot 10^{-6}$			
$\hat{d}_{m,1}$ [-]	100.00	50.00	50.00	40.00
$\hat{d}_{m,2}$ [-]	70.71			
γ_1 [d^{-1}]	0.40			
γ_2 [d^{-1}]	2.89			
h_1 [-]	2.29			
h_2 [-]	2.20			
α_1 [-]	$1.95 \cdot 10^{-2}$			
α_2 [-]	$2.29 \cdot 10^{-2}$			
β [-]	-0.08			

Supplementary Table S5. Parameter values used in the four computer simulations of our biomechanistic model of breast cancer response to NAC based on doxorubicin and cyclophosphamide (subindices 1 and 2, respectively) shown in Figure 7 in the main text. The values of the rest of the parameters in the model, which were not included in the sensitivity analysis, are provided in Section 2.3.4 of the main text.

Parameters [units]	Model simulations in Figure 11			
	Panel A	Panel B	Panel C	Panel D
D_0 [mm^{2}d^{-1}]	$3.10 \cdot 10^{-4}$			
ρ_0 [d^{-1}]	$2.50 \cdot 10^{-2}$			
$\hat{E}_1$ [-]	-1.50	-0.65	-0.80	-0.30
$\hat{E}_2$ [-]	-1.50	-0.65	-0.80	-0.30
C_1 [M]	$2.00 \cdot 10^{-9}$			
C_2 [M]	$1.35 \cdot 10^{-6}$			
$\hat{d}_{m,1}$ [-]	100.00	50.00	50.00	40.00
$\hat{d}_{m,2}$ [-]	100.00	50.00	50.00	40.00
γ_1 [d^{-1}]	0.77			
γ_2 [d^{-1}]	0.13			
h_1 [-]	2.57			
h_2 [-]	0.62			
α_1 [-]	$2.51 \cdot 10^{-1}$			
α_2 [-]	$7.18 \cdot 10^{-1}$			
β [-]	-0.01			

Supplementary Table S6. Parameter values used in the four computer simulations of our biomechanistic model of breast cancer response to NAC based on paclitaxel and carboplatin (subindices 1 and 2, respectively) shown in Figure 11 in the main text. The values of the rest of the parameters in the model, which were not included in the sensitivity analysis, are provided in Section 2.3.4 of the main text.
